# Extreme Ultraviolet Photoresponse of Organotin-Based Photoresists with Borate Counteranions

**DOI:** 10.1021/acsami.4c08636

**Published:** 2024-08-05

**Authors:** Quentin Evrard, Najmeh Sadegh, Simon Mathew, Ed Zuidinga, Benjamin Watts, Maximilian Paradiz Dominguez, Angelo Giglia, Nicola Mahne, Stefano Nannarone, Akira Nishimura, Tsuyoshi Goya, Takuo Sugioka, Michaela Vockenhuber, Yasin Ekinci, Albert M. Brouwer

**Affiliations:** † Advanced Research Center for Nanolithography ARCNL, Science Park 106, 1098 XG Amsterdam, The Netherlands; ‡ van’t Hoff Institute for Molecular Sciences, University of Amsterdam, P.O. Box 94157, 1090 GD Amsterdam, The Netherlands; § Paul Scherrer Institute, Forschungsstrasse 111, 5232 Villigen PSI, Switzerland; ∥ CNR-IOM—Istituto Officina dei Materiali, National Research Council of Italy, Strada Statale 14 km 163, 5, Basovizza, Trieste 34149, Italy; ⊥ Nippon Shokubai, 5-8 Nishi Otabi-cho, Suita, Osaka 564-0034, Japan

**Keywords:** extreme ultraviolet lithography, tin-based photoresist, tin-oxo-hydroxo cage, Inorganic−organic hybrid photoresist, metal-based photoresist

## Abstract

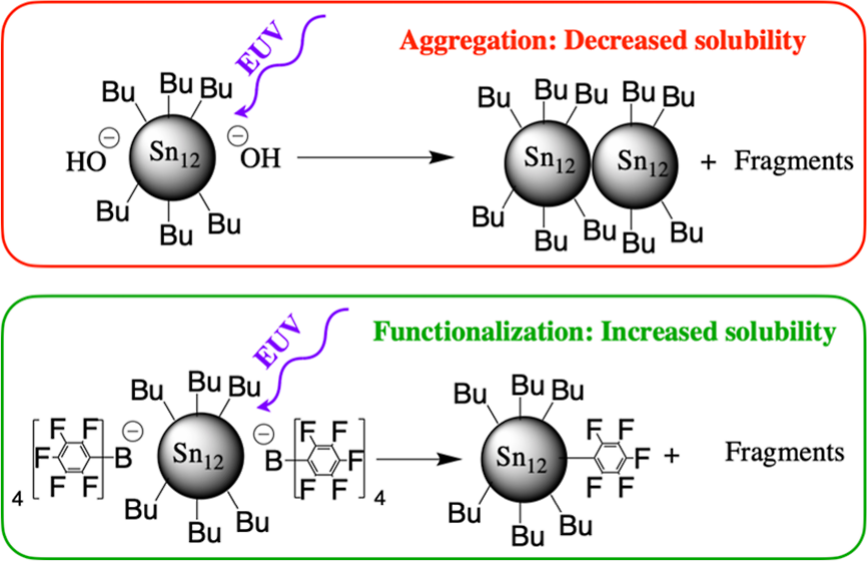

Organometallic tin-oxo-hydroxo cage compounds offer a promising photoresist platform for extreme ultraviolet photolithography (EUVL). Their reactivity is dominated by the facile breaking of the tin–carbon bonds upon photon or electron irradiation. As the cage is dicationic, it exists as a complex with anions for charge compensation. In the present work, we explore the *n*-butyltin-oxo cage with two tetrakis(pentafluorophenyl)borate counteranions (TinPFPB). In contrast to the small counterions that are typically used, the bulky PFPB anion absorbs a substantial fraction (∼30%) of the impinging EUV radiation (13.5 nm, 92 eV), and it has its own reactivity upon photoionization. When thin films of the complex are irradiated with EUV radiation at low doses, a positive-tone development is possible, which is rather unique as all other known tin-oxo cage resists show a negative tone (cross-linking) behavior. We propose that the initial positive tone behavior is a result of the chemical modification of the Sn cluster by fragments of the borate anions. For comparison, we include the tetrakis(*p*-tolyl)borate anion (TB) in the study, which has similar bulkiness, and its complex with the *n*-butyltin-oxo cage (TinTB) shows the usual negative tone EUV resist behavior. This negative-tone behavior for our control experiment rules out a hypothesis based purely on the steric hindrance of the anion as the cause of the different EUV reactivity.

## 1 Introduction

The increased need for both more powerful and more energy efficient electronic devices fuels research for better lithographic techniques. After the invention^1^ and development^2^ of excimer lasers, their application to photolithographic purposes was rapidly adopted.^3^ For more than 15 years, the electronics industry relied on ArF (193 nm) lithography for the highest resolution in computer chip manufacturing,^4,5^ improving the process by using immersion lithography techniques,^6,7^ and combining this with multiple patterning techniques to decrease the reachable feature size even further.^8,9^ To print smaller sizes in a single step extreme ultraviolet (EUV) lithography has been introduced, which uses a wavelength of 13.5 nm.^10^ In parallel to the optical and mechanical improvements of exposure tools, the need arises for better photoresists materials more suited to higher resolution, sensitivity, and pattern fidelity.^11^ As features get smaller, the photoresist needs to be thinner because of the decreased depth of focus^12^ in high NA EUV lithography and to decrease the aspect ratio of the structures to avoid pattern collapse. With thinner resist films, the need arises for higher etch resistance to allow for pattern transfer and for a higher absorption factor to make the most efficient use of the expensive EUV photons. This field of research has been largely dominated by organic resists^13−17^ and chemically amplified organic resists (CAR).^18−23^ Although specially adapted CAR can still meet the current requirements of EUV lithography, the organic photoresist approach is less suited to match with industrial requirements for future technology nodes.^24−28^

To alleviate the inherent issues of organic photoresists such as relatively low etch resistance^29,30^ and their lower EUV absorption cross-section,^31,32^ new hybrid materials containing metals^33−39^ or nanoparticle approaches^40,41^ have been investigated. The addition of inorganic atoms can provide both a higher EUV absorption cross-section and higher etch resistance in thin films.^42−44^ A particular effort has been centered around Sn_12_-oxo-hydroxo cages,^34,44−49^ which exhibited promising patterning properties upon initial investigation.^34^ Importantly, Sn has a large absorption cross-section at 92 eV (for several tin cages, α = −ln *T* ≈ 12 μm^–1^),^50^ which makes it possible to capture ∼25% of the impinging EUV photons in a 25 nm thick film of tin-oxo cages with small counterions.

**Scheme 1:**
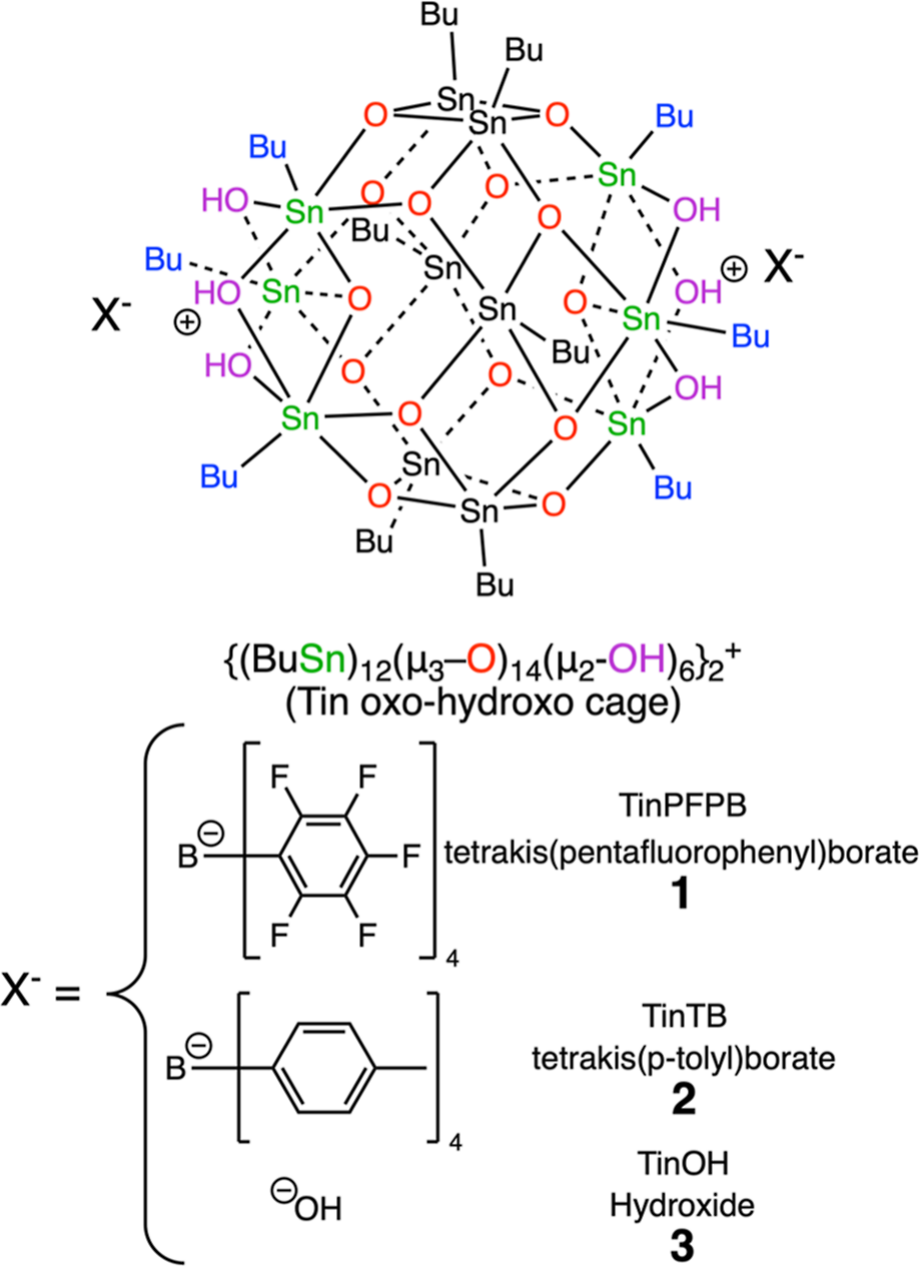
Schematic Structure of *n*-Butyl-Sn_12_-Oxo-Hydroxo Cages and Anions Used in the Present Work

The tin cages, as shown in Scheme 1, are dications,^51^ and to balance the charge, they are associated with anions that can be readily exchanged and used to tune the properties of the system.^52,53^ When the anions are small, the relative concentration of Sn atoms in the photoresist films is high, which is beneficial for a high EUV absorption cross-section. The role of the anion has been explored to some extent. Cardineau et al.^34^ concluded that for a series of carboxylate anions, the size was important: larger anions reduced the sensitivity due to the dilution of the tin atoms in the film, which reduced the EUV photon absorption per unit of film thickness. Haitjema et al.^54^ and Sadegh et al.^55^ proposed that the anions (sulfonates and acetate) play an active role in the photolithographic reaction mechanism because they act as nucleophiles and attack the tin cage after photoionization and the loss of a butyl radical. Ma et al.^56^ revealed in a computational study that OH^–^ as a counteranion can act as a base and abstract a proton from one of the bridging OH groups. Bespalov et al.^47^ noted that the tin-oxo cages with OH counteranions (**3**, “TinOH”) were unstable in silico and to retain its structure at different levels of theory required adding hydrogen bonding units (water molecules) in the model to “tame” the basicity and nucleophilicity of OH^–^. In a seminal study of TinOH, Banse et al.^51^ observed that isopropanol was built into the crystal structure and that the crystals would become translucent, amorphous, and insoluble in C_6_D_6_ or CD_2_Cl_2_ upon the loss of cosolvent.

In the present work, we study the *n*-butyltin-oxo cage with tetraarylborate counteranions, specifically tetrakis(pentafluorophenyl)borate (**1**, TinPFPB) and tetrakis(*p*-tolyl)borate (**2**, TinTB). These anions are chemically relatively inert: neither basic nor nucleophilic. As these anions take up a considerable volume, the EUV absorption of the thin film is expected to be reduced. The molecular absorption cross-sections can be estimated rather reliably using tabulated atomic cross-sections.^31^ To calculate the absorption coefficient of the thin film (α, commonly expressed in units of μm^–1^), the density should be known. For several *n*-butyltin-oxo cages with small counterions, this was found to be ∼1.9 g cm^–3^. For TinPFPB **1**, a crystal structure was determined (see below), from which we derive the density as ρ = 2.131 g cm^–3^. For **2**, we estimate a density of ∼1.7 g cm^–3^ based on computed molecular volumes and the experimental data for **1** and **3** (see Supporting Information). Using these densities, we can estimate absorption coefficients at 92 eV as α = 14.7 μm^–1^ for TinPFPB **1** and α = 10.5 μm^–1^ for TinTB **2** (Table 1). Compared to α = 12.7 μm^–1^ for TinOH **3** (from atomic cross-section and crystal structure data, including 4 iPrOH solvent molecules^51^), we see the expected reduced cross-section for TinTB, but for TinPFPB, the loss of absorption due to dilution of tin is compensated by the presence of the 40 fluorine atoms, which have the highest cross-section of the second row elements along with a relatively small van der Waals radius. The result is that for compound **1** of the photons absorbed at 92 eV, 49% will lead to initial ionization of Sn and 29% to ionization of F in addition to 21% valence ionization of C and O.

**Table 1:** Absorption Properties at 92 eV^b^

compound	α (μm^–1^)	*f*_CHB_	*f*_O_	*f*_Sn_	*f*_F_
TinPFPB (**1**)	14.7	0.132	0.093	0.486	0.289
TinTB (**2**)	10.5^a^	0.201	0.129	0.671	
TinOH (**3**)	12.7	0.124	0.175	0.701	

a Using estimated density, see Supporting Information for additional details.

b α is the absorption factor based on tabulated cross-sections and experimental densities and *f*_X_ is the fraction of photons absorbed by element(s) X.

The choice of the anion was made by keeping the high EUV absorption cross-section requirement in mind and, as such, to offset the loss of EUV absorption caused by the addition of organic components by using fluorinated anions.^31^ The photochemistry of tetraphenyl borates has been extensively investigated for the 200–350 nm irradiation range.^57−60^ Rearrangement and fragmentation products have been identified. To the best of our knowledge, the effect of 13.5 nm (EUV) excitation or other ionizing radiation has not been studied. Photochemical reactions with electron acceptors, however, cause the decomposition of tetraarylborates via electron transfer in a pathway that is similar to photoionization.^60^ The solubility switch of the tin cage resist films upon exposure to EUV relies on the cleavage of Sn–C bonds, causing the loss of the butyl radicals.^54^ Subsequently, cross-linking of the tin clusters is thought to occur, rendering the material insoluble.^34^ Additionally, experiments done with UV and VUV exposure in the gas phase^54^ show that homolytic cleavage of tin–carbon bonds is observed for all photon energies above the onset of electronic absorption around 5 eV (250 nm) that led to photoproducts, which have lost one or two butyl groups. After the double butyl loss, a structural rearrangement was proposed to recouple the unpaired electrons of the Sn-centered radicals connected to the OH groups.^54^ Furthermore, covalent binding of the sulfonate counteranion to the tin cluster used in that work is expected to occur.^54^ The chemistry following ionization (at photon energies *E*_ph_ > 12 eV in the gas phase^54^ and 8 eV in the solid phase^55^) also starts with Sn–C bond cleavage. In the gas phase, excess thermal energy allows further fragmentation^54,61^ but in the solid state, the product of ionization and butyl loss is a closed shell molecule that will not easily lose further butyl groups.

## 2 Experimental Section

Experimental methods are briefly described here. Further details are given in the Supporting Information.

The *n*-butyltin-oxo hydroxo cage TinOH **3** was synthesized according to known procedures.^52^ The borate derivatives were prepared from **3** by anion exchange. Synthetic details and the spectroscopic characterization of the products can be found in the Supporting Information. Single crystals of **1** were obtained from a 50/50 mixture of toluene and trifluorotoluene, enabling analysis via single crystal X-ray diffraction. After adding hydrogen atoms at calculated positions to the obtained crystal structure, the resulting model was optimized using the B3LYP hybrid density functional method with the LANL2DZ basis set. From the model thus obtained, a model of **2** was derived by replacing the substituents on the benzene rings and optimized in the same way to estimate the density of **2** by comparison with that of **1**.

Thin films of the compounds were prepared by spin coating on silicon wafers or silicon nitride membranes. In some cases, the wafers, which have a native oxide layer, were pretreated with hexamethyldisilazane to create a hydrophobic surface.^62^ The thin films were characterized by means of atomic force microscopy (AFM), infrared spectroscopy, X-ray photoemission spectroscopy (XPS), and absorption spectroscopy. The films were exposed to 92 eV radiation and studied again using spectroscopic methods or developed using a suitable solvent to study the solubility switching behavior. After the dissolution of the whole exposed film, mass spectrometry analysis was applied to gain insight into the photoproducts formed.

## 3 Results and Discussion

### 3.1 Structure of TinPFPB

**Figure 1:**
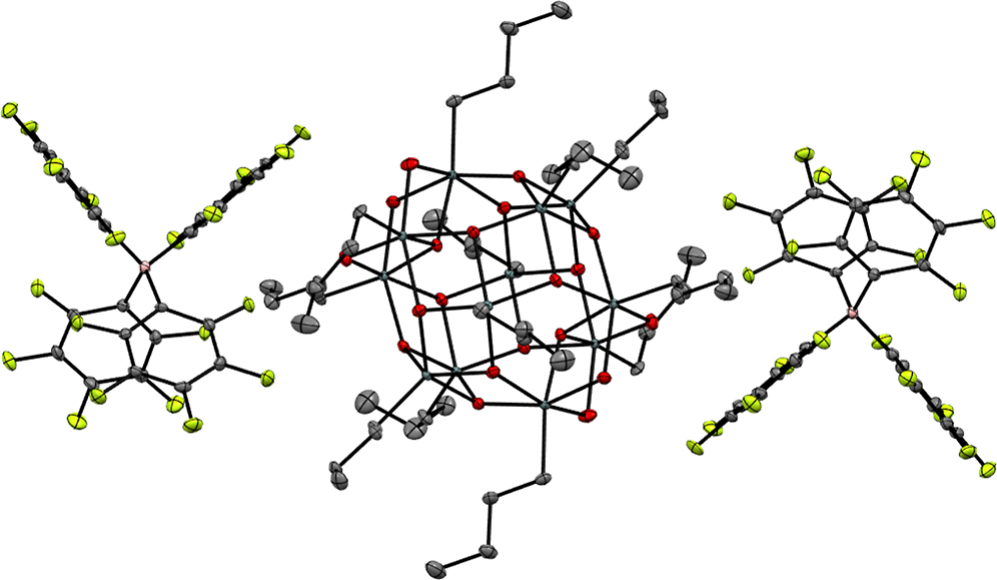
Structure of TinPFPB **1** obtained via single crystal X-ray diffraction at 100 K.

Single crystal X-ray diffraction allowed us to resolve the structure of TinPFPB **1** (Figure 1), with full details in the Supporting Information. The structure of the dicationic core is in agreement with previously reported structures for Sn_12_ clusters:^51^ a central Sn_6_O_12_ ring composed of 5-coordinated Sn atoms bridged by μ_3_-O atoms, and on both sides of it, a “cap” structure with three 6-coordinated Sn atoms that are connected via three μ_2_–OH groups and one μ_3_-oxygen. All Sn atoms have one butyl group pointing outward, and the two PFPB anions each have one pentafluorophenyl ring facing their respective 3 μ_2_–OH moieties. The minimum distance between two butyl-Sn_12_-oxo-hydroxo cages in this structure is 10.7 Å as opposed to the 7.9 Å observed in the structure of TinOH.^51^

### 3.2 NMR Spectroscopy

The ^1^H, ^13^C, ^119^Sn, ^11^B, and ^19^F NMR spectral data of **1** and **2** agree with the expected structures (Figures S5–S22). When comparing the ^1^H NMR spectra of **1** and TinOH **3** (Figure 2) in CDCl_3_, a large difference in the chemical shifts of the protons of the *n*-butyl groups is apparent.

**Figure 2:**
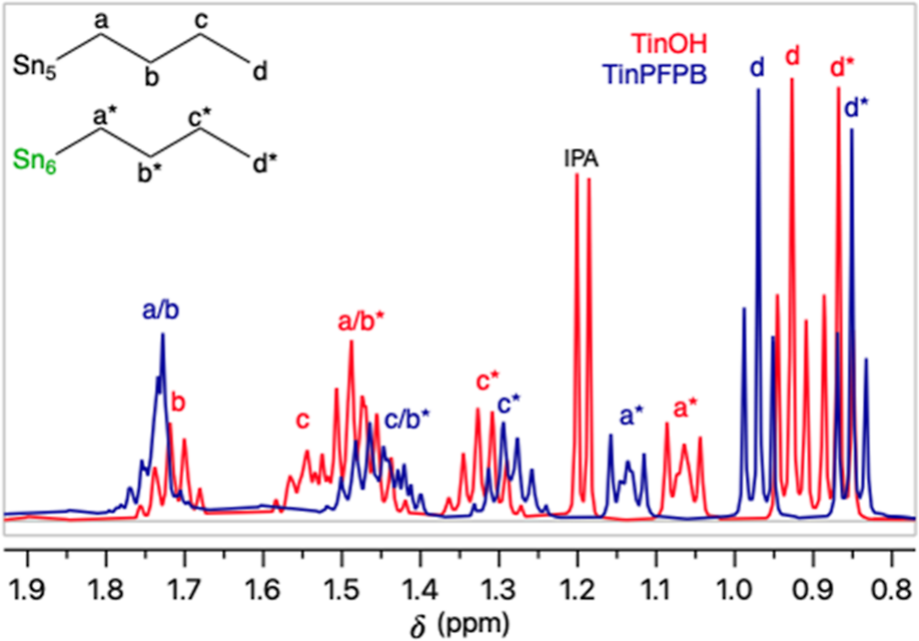
Partial ^1^H NMR spectra (400 MHz, CDCl_3_) of **1** (blue) and **3** (red), Sn_5_ are 5-coordinated, and Sn_6_ are 6-coordinated atoms (see Scheme 1); IPA = isopropanol.

Notably, the chemical shifts of the terminal CH_3_ groups of the *n*-butyl groups of the caps (d*) and those of the belts (d) are further apart in **1** than in **3**. The difference most likely arises from the differences in the shielding by the aromatic rings of the borate anions. In methanol-*d*_4_, a polar solvent, the ions are dissociated and the differences between the ^1^H NMR spectra of **1** and **3** are small (Figure S25).

### 3.3 Exposure of Thin Films to EUV Irradiation

**Figure 3:**
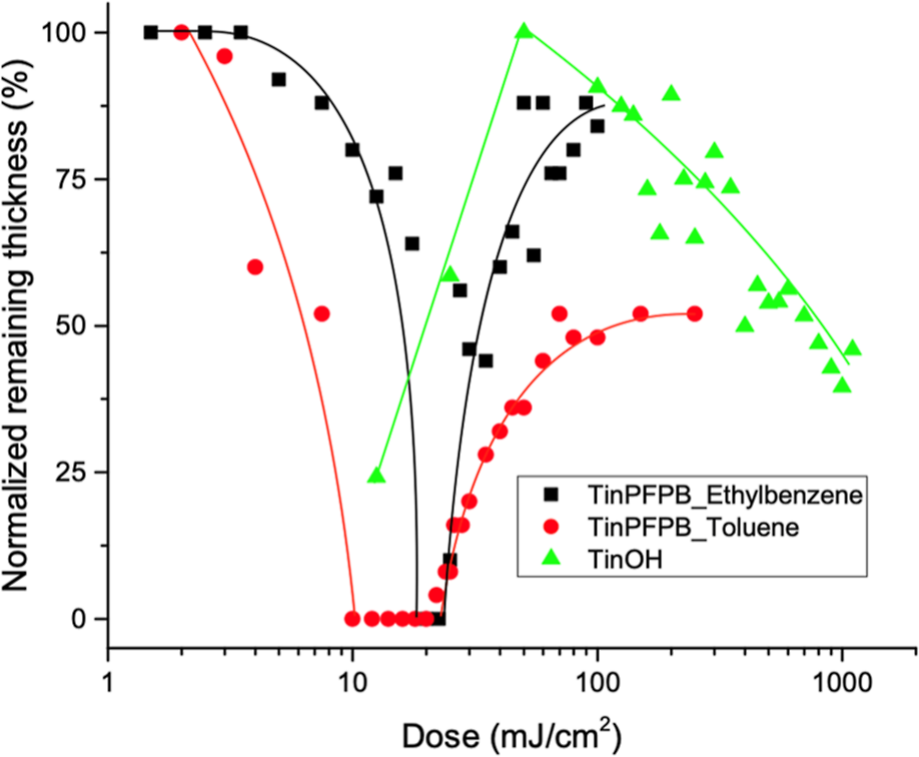
Measured remaining thickness of films of TinPFPB **1** (black: developed with ethylbenzene; red: developed with toluene) and TinOH **3** (green: developed with iPrOH/H_2_O mixture 2:1^45^) exposed to variable EUV doses (lines are just a guide for the eye).

One of the key parameters in the evaluation of a new photoresist is the measurement of the dose required to achieve a switch of the solubility of the exposed vs unexposed areas stemming from a chemical change in the photoresist. From previous work,^47,54,56^ the main mechanism of the solubility switch is thought to be the homolytic cleavage of Sn–C bonds, leading to a reduction of number of butyl groups attached to one cage coupled with a potential cross-linking between two tin centers further decreasing the solubility of the obtained material. These mechanisms led to a decrease in solubility due to EUV irradiation, which corresponds to negative tone photoresist behavior. This behavior can be quantitatively assessed by the measurement of the remaining thickness of photoresist after the development versus EUV dose received by the sample via AFM, as shown in Figure 3. Data for TinOH are included as an example of the standard behavior of tin-oxo-hydroxo cages, in which the remaining thickness increases upon an increase of EUV dose followed by a slow decline in thickness due to further degradation of the material due to overexposure. Surprisingly, for TinPFPB **1**, the observed behavior is drastically different, with first a decrease of remaining film thickness with an increase of EUV dose, reaching a plateau where no photoresist remains on the exposed surface between 10 and 20 mJ cm^–2^, followed by a gradual increase in the remaining thickness for a higher exposure dose. This positive tone photoresist behavior was unexpected, and although the dual-tone nature of the tin oxo-hydroxo cage system has been previously reported,^46^ very little is known concerning the mechanistic reasons for such change. To that end, several additional experiments listed below were performed to provide a better understanding of the chemical solubility switch of TinPFPB **1**.

### 3.4 Infrared Spectroscopy

#### 3.4.1 Infrared Spectra of TinPFPB

Infrared spectra of TinPFPB recorded for the bulk material via ATR spectroscopy (Figure S23) show the expected absorption bands stemming from the butyl groups (2953 cm^–1^ for CH_3_ antisymmetric stretching and 2870 cm^–1^ for symmetric stretching; 2920 cm^–1^ for CH_2_ antisymmetric stretching and 2855 cm^–1^ for symmetric stretching). The sharp peak at 3636 cm^–1^ corresponds to the OH groups present on the caps of the tin cluster and indicates that they do not have a hydrogen bonding interaction with the anion.^63^ In tin-oxo cage compounds with small hydrogen bond accepting counterions such as OH^–^ or OAc^–^, a sharp feature is not found, and the OH stretching vibrations give rise to a broad band in the 2900–3700 cm^–1^ range. In the fingerprint region, characteristic bands from the phenyl rings of the PFPB anion are seen at 1641, 1515, and 1468 cm^–1^. Stretching vibrations due to C–F and C–B bonds appear at 1270 and 1086 cm^–1^, respectively.

#### 3.4.2 Infrared Spectroscopy of Exposed TinPFPB 1 Films

**Figure 4:**
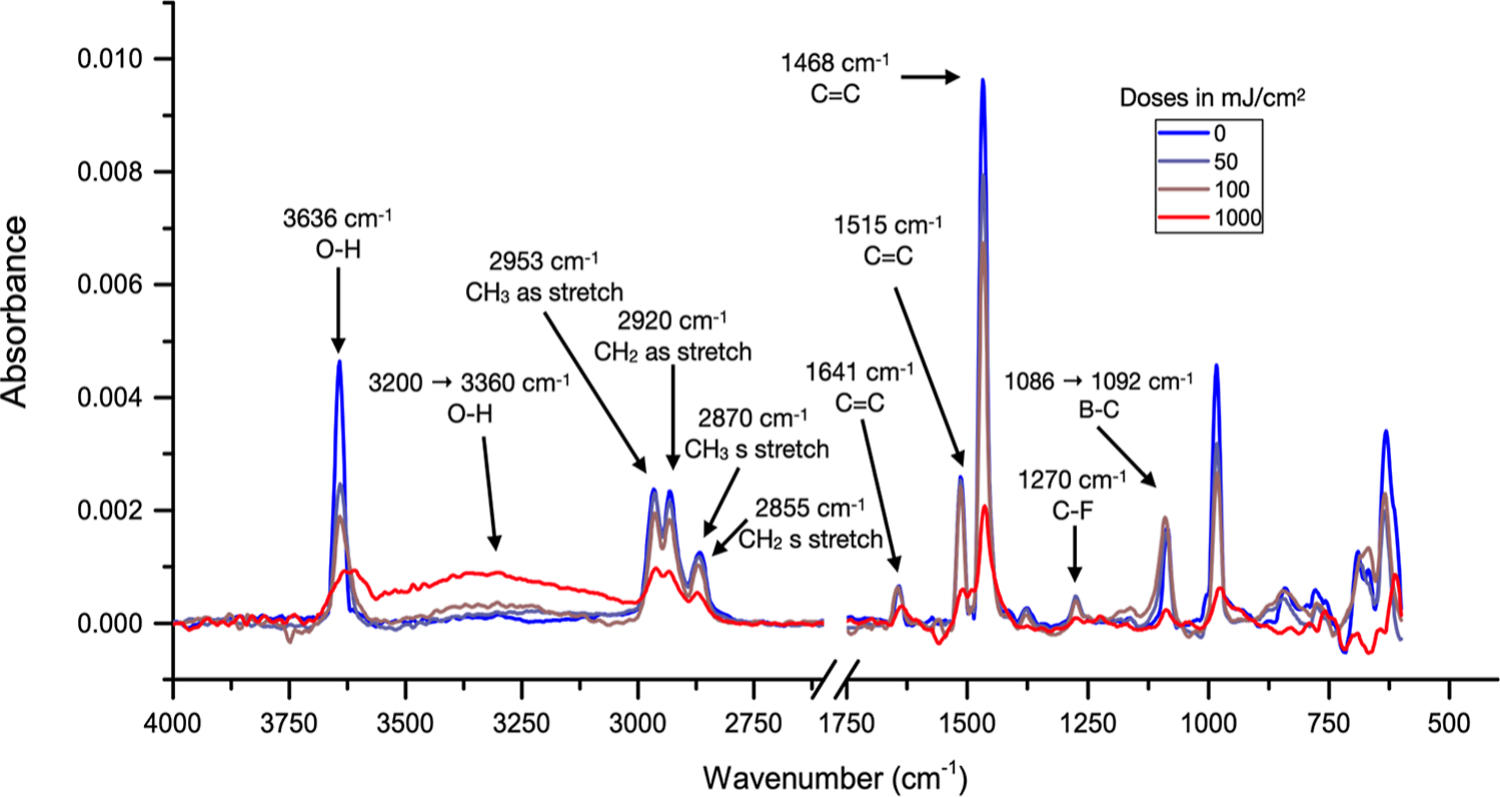
Infrared spectra of film of TinPFPB **1** exposed to EUV at doses ranging from 0 to 1000 mJ cm^–2^. (Full set of irradiation doses is shown in Figure S24.)

Thick films of TinPFPB (∼80 nm) were prepared on gold substrates (Supporting Information, Section S3) and then irradiated at the XIL-II beamline (SLS-Paul Scherrer Institute, Switzerland) on 500 μm wide squares at different EUV doses. The sample was then measured in reflectance mode using a FT-IR microscope and led to the spectra, as shown in Figure 4 below. The unexposed film spectrum matches well with the spectrum of the bulk (Figure S23), which indicates that no drastic chemical change occurred during the spin coating procedure, apart from the expected signature of the anion in the 1000–1700 cm^–1^ region and of the *n*-butyl chains of the tin cage in the 2850–2950 cm^–1^ region. The sharp peak at 3636 cm^–1^ observed on the unexposed material, interestingly, shows a sharp 30% decrease with a dose as low as 50 mJ cm^–2^ as well as a broadening, indicating that the environment around the caps is perturbed. Another indication of the perturbation of the OH of the caps stems from the appearance of a very wide OH band in the 2900–3600 cm^–1^ region. The B–C band from the anion at 1086 cm^–1^ also shifts toward 1092 cm^–1^, which, combined with the mass spectrometry results (vide infra), indicates that it is likely that some OH are recombined or interacting with photoproducts of the anion degradation.

### 3.5 In Situ X-ray Photoelectron Spectroscopy of Exposed TinPFPB 1 Films

XPS spectra of thin films of **1** were collected at the BEAR beamline^64^ (see Supporting Information for details), as shown in Figure S26. Two peaks are present at the C 1s edge. The binding energy of 288 eV corresponds to the carbons of the PFPB anion and the binding energy of 285.4 eV corresponds to the carbons of the butyl chains of the tin cage.^65^ The area of each peak being proportional to the number of species present at the surface of the material, we can observe a decrease of the signal at 285.4 eV from the butyl chains of 26% at 100 mJ cm^–2^. Meanwhile, the peak area stemming from the PFPB anion sees an increase of 27% as well as a widening shifting toward lower binding energy. It must be noted that as the measurements have been performed under vacuum (≈7 × 10–9 mbar), no in situ development was done, and only volatile species may leave the material during the exposure. This clearly indicates that butyl chains are still cleaved by the EUV exposure as for other Sn cage materials. Although the binding energy of its signal is reduced by only 0.2 eV, the anion itself is likely also chemically modified by the EUV exposure, as seen from the signal broadening. The relative increase of signal from the anion comes mainly from the removal of *n*-butyl chains and hence a reduction of its screening effect: as butyl groups leave the surface of the film, the density of fluorine-bound carbon near the surface increases, and thus a stronger signal may be expected.

Comparing these results with the infrared spectroscopy in 3.4, it must be noted that for the butyl chain bands in the 2855–2950 cm^–1^ region, the decrease of overall band signal is only 5% at 50 mJ cm^–2^ and 13% at 100 mJ cm^–2^, which is less than the 26% at 100 mJ cm^–2^ found via XPS analysis. However, as opposed to very surface sensitive XPS, IR spectroscopy effectively probes the entire film composition. Moreover, outgassing products (butane, butene, and octane) might be retained deeper inside the film.

**Figure 5:**
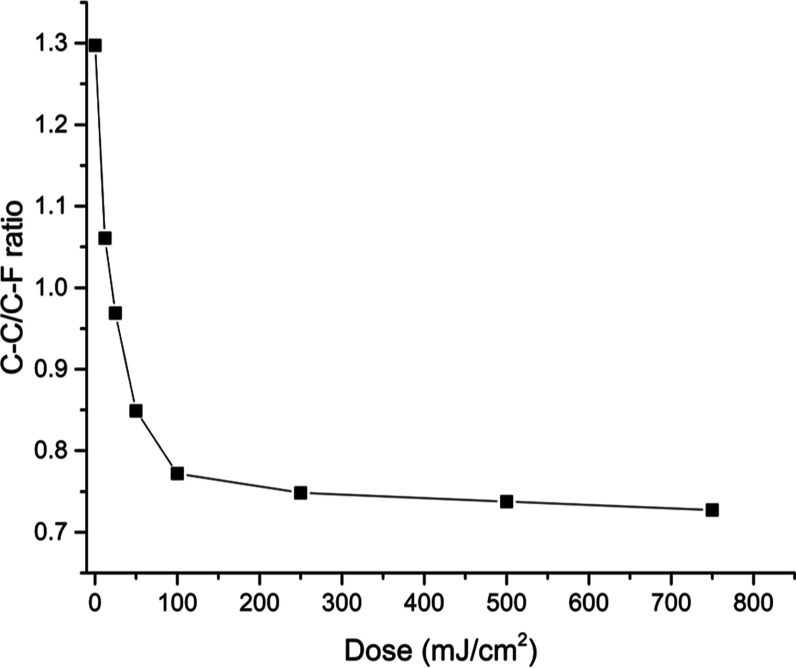
C 1s XPS ratio of the signal of butyl chains to that of the C atoms in B(PFP)_4_ anion of TinPFPB **1**.

Figure 5 shows the ratio between the area of the peaks stemming from the butyl chains over those of the B(PFP)_4_ anion and the EUV exposure dose. This graph shows a sharp decrease from 0 to 100 mJ cm^–2^ from 1.3 (close to the theoretical value of 1.4) to a value of 0.77 and then a plateau is reached for higher doses, showing that most of the major chemical changes are happening within the first 100 mJ cm^–2^.

This difference in apparent bleaching could be due to a difference in volatility between the reaction products of EUV exposure from the tin cage (*n*-butyl groups) and from the anion (PFPB), as well as the different chemical pathways for both byproducts. To delve deeper into the reactivity of the TinPFPB **1** system, mass spectrometric analysis has been performed on the dissolved materials obtained from development, and the results are shown below in Section 3.7.

### 3.6 X-ray Absorption Spectroscopy

**Figure 6:**
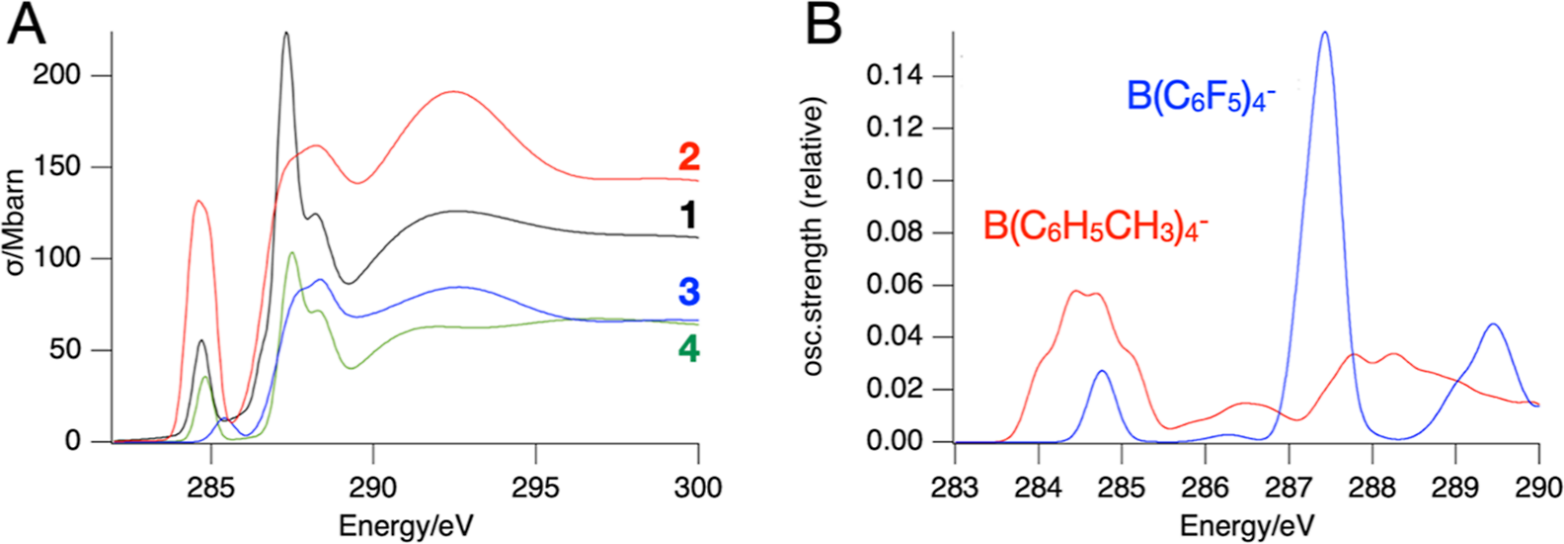
Absorption spectra at the carbon K edge. (A) Experimental spectra (smoothed; see Figure S1–S3 for raw data) of **1**, **2**, **3**, and tetramethylammonium tetrakis[pentafluorophenyl] borate **4**. (B) Calculated spectra of borate anions, shifted to match the low-energy peak at 284.7 eV.

Soft X-ray absorption spectra (XAS) were measured at the carbon and fluorine K-edges of spin-coated films of the tin-oxo cages on the SiN membranes. In contrast to XPS, these measurements, which are carried out in transmission mode, probe the entire film thickness. Thus, they are not very sensitive to possible surface contamination. Figure 6 shows the spectra at the C K-edge of **1**, **2**, **3**, and tetramethylammonium TinPFPB. The spectra were normalized by setting the absorbance at 340 eV equal to the theoretical cross-section for the photoionization of C 1s at that energy.

A characteristic feature of the XAS of TinOH **3** is the low-energy absorption band at 285.4 eV^61^ due to the C 1s → lowest unoccupied molecular orbital (LUMO) (σ*) transition. In the phenylborate derivatives, a much stronger absorption occurs in this energy range that can be attributed to the C 1s → π* transitions of the aromatic rings. The band peaks at 284.7 eV for **1**. For **2**, it is at the same energy but is clearly stronger and broader. In both cases, the C 1s → σ* transitions of the S*n*-butyl groups are not resolved as a separate peak. A second strong band in **1** at 287.3 eV can be largely attributed to C 1s → π* transitions. Calculations of the XAS of the borate anions using the transition potential method^66−68^ reveal that the low-energy peak in **1** is due to excitations involving the C 1s orbitals of the C atoms bonded to B, while the higher-energy peak stems from transitions involving the C 1s orbitals of the C atoms bonded to F, which have a higher binding energy. For the tolylborate anion, all C atoms have similar binding energies, and the transitions to the low-lying π orbitals occur at similar energies. The fluorinated compounds **1** and **4** were also measured by XAS at the fluorine K-edge (Figure S27).

**Figure 7:**
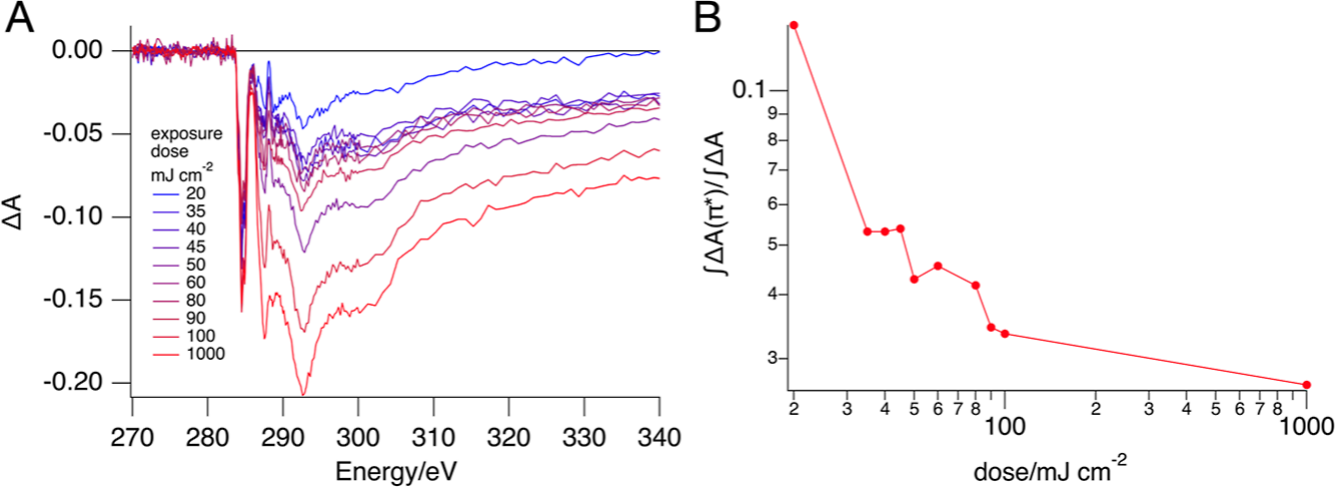
(A) Bleaching of a thin film of **2** at the indicated EUV doses. (B) Ratio of the bleaching of the C 1s → π* band (283–286 eV) to the total bleaching.

For tolylborate derivative **2**, we monitored the spectral changes during EUV exposure by observing the change in transmission of exposed and unexposed regions (Figure 7). This experiment demonstrates that at the initial stages of the conversion, the band of the C 1s → π* transition at 284.7 eV is more strongly bleached than the broad band at higher energies, which contains contributions from all carbon atoms.

### 3.7 Mass Spectrometry of Exposed TinPFPB 1 Films

**Figure 8:**
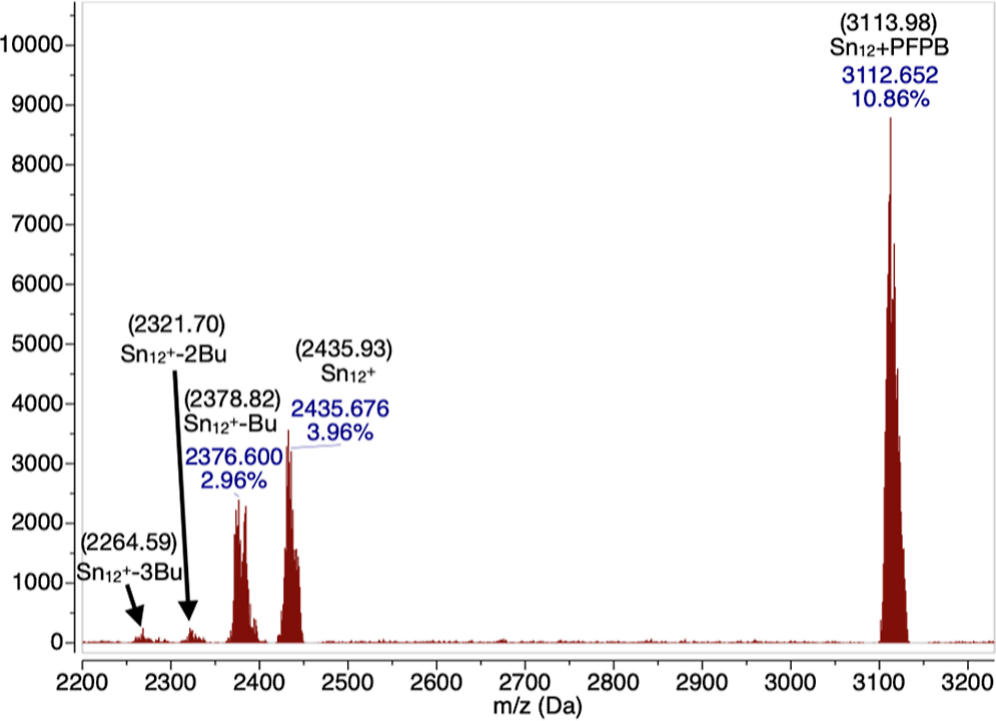
Positive charge mass spectrometry of bulk TinPFPB **1** obtained by electrospray ionization, numbers in parentheses are calculated values for each fragment.

**Figure 9:**
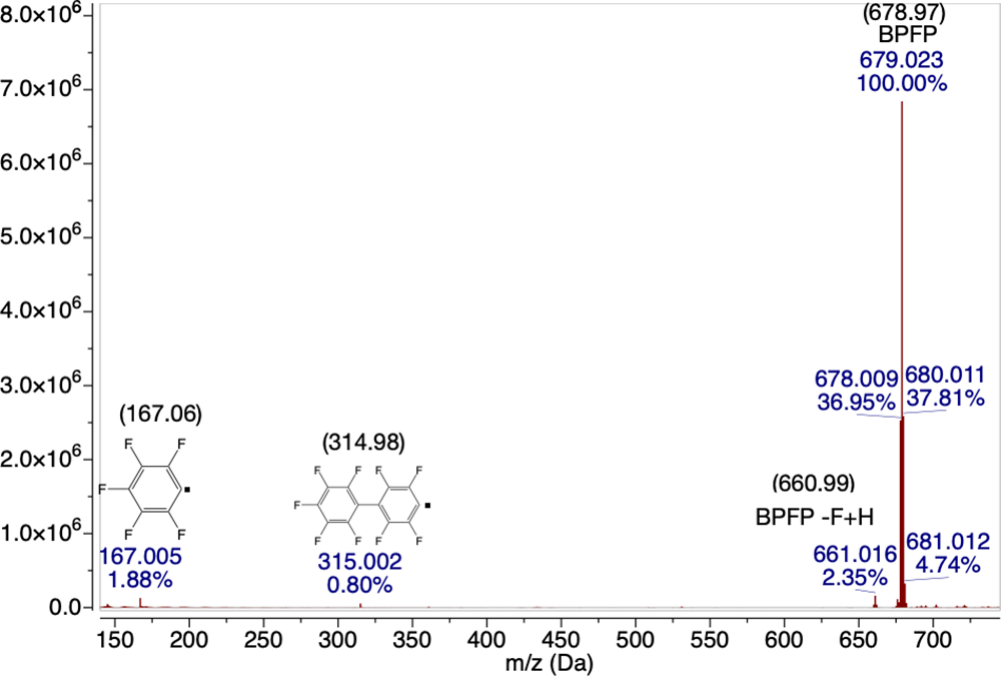
Negative charge mass spectrometry of bulk TinPFPB **1** obtained by electrospray ionization.

To get a better understanding of the positive tone reactivity of TinPFPB **1**, we performed mass spectrometry on material dissolved by ethylbenzene (developer) on films exposed to a 20 mJ cm^–2^ EUV irradiation dose. As a reference, the mass spectrometry of the bulk material has been recorded using ethylbenzene for both positive and negative ions, and the resulting electrospray ionization mass spectra are shown in Figures 8 and 9. For the positive charge mass spectra, even with mild ionization conditions, a degradation of the tin cages has been observed, with a *m/z* of 2435 Da coming from a tin cage having one positive charge instead of two, followed by 3 peaks of lower *m/z*, indicating the loss of 1, 2, and 3 butyl groups, respectively. The presence of a complete tin cage with one PFPB adduct is also visible as the main species, with a *m/z* of 3115 Da. This kind of behavior is similar to what has been previously reported on similar tin cage systems.^69^ Due to the large number of tin isotopes naturally present in the sample, a finer analysis on the positive ion mass spectra could not be performed.

The negative ion mass spectra of the bulk TinPFPB **1** material give mainly the expected peak from the PFPB anion at 679 Da, but it also reveals small amounts of byproducts, such as a species with a hydrogen atom replacing a single fluorine from the anion, leading to a *m/z* of 661 Da. The mass spectrum also reveals B(PFP)_3_F, PFP anion, and PFP dimer radical anion are present (Figures 9 and S5), which is similar to what has been reported for UV induced reactions of tetraarylborates such as B–C cleavage and formation of diaryl species.^57^

**Figure 10:**
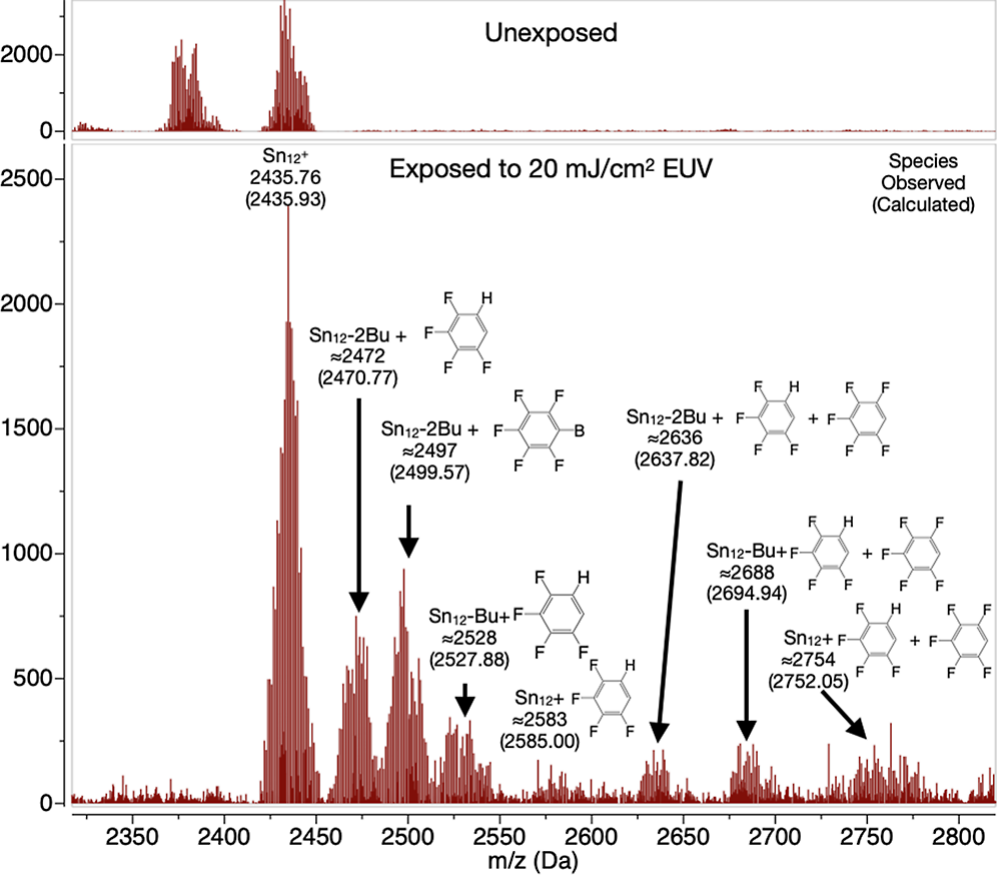
Positive charge mass spectrometry of films of TinPFPB **1** exposed to 20 mJ cm^–2^ EUV and developed with ethylbenzene obtained by electrospray. Top inset is unexposed TinPFPB **1** in the same conditions for reference. Sn_12_–Bu and Sn_12_-2Bu refer to a tin cluster having lost one and two butyl moieties, respectively.

The mass spectra were collected from TinPFPB **1** films exposed to 20 mJ cm^–2^ EUV irradiation and then dissolved using ethylbenzene as a developer. The solution was then transferred to the mass spectrometer and led to the spectra, as presented in Figure 10. The most striking result is that none of the previously observed lighter species due to the loss of butyl chains in the bulk sample are present below the Sn_12_^+^ cage *m/z* of 2435 Da. Instead, only heavier species that are a combination of the addition of either a single pentafluorophenyl and/or a tetrafluorophenyl with the loss of zero, one, or two butyl chains are observed. Interestingly, in the Sn_12_^2+^ part of the spectra, both Sn_12_^2+^ and Sn_12_^2+^ with one less butyl group can be observed (Figure S8). These results indicate that not only is the Sn_12_ cage reactive but also the PFPB and its EUV decomposition products are reactive and can readily attach to the tin cage itself. These chemical changes are likely to modify the solubility of the tin cages enough to give rise to a solubility switch and thus positive tone behavior at the 20 mJ cm^–2^ EUV dose.

### 3.8 Reaction Mechanism

**Figure 11:**
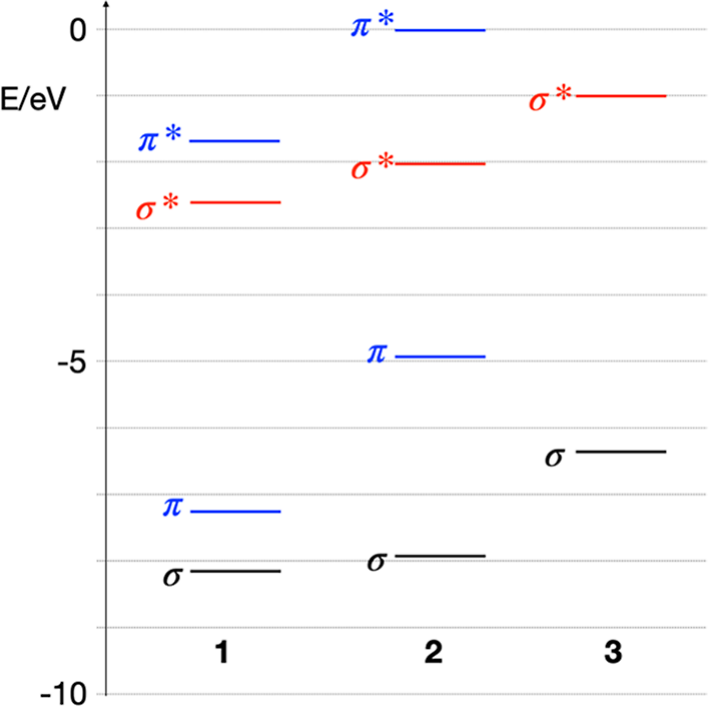
Orbital energy levels (B3LYP/LANL2DZ) of highest occupied σ and π orbitals, and lowest unoccupied σ* and π* orbitals for tin cages **1**, **2**, and **3**.

Quantum-chemical calculations give insight into the molecular electronic structure and expected reactivity. For the tin-oxo cages with small counterions (OH^–^ and OAc^–^), the highest occupied molecular orbitals (HOMOs) have Sn–C σ bonding character, and LUMO has σ* character. Both photoionization and electron capture of the tin-oxo cages readily lead to the cleavage of Sn–C bonds.^34,47,54,61,70^ For the borate derivatives studied in this work, the HOMOs are located on the borate anion. The computed orbital energy levels are shown in Figure 11.

Graphical representations of the corresponding molecular orbitals are in the Supporting Information (Figure S4). The first step in EUV photochemistry is the ionization of the substrate. The primary photoelectron will generate 2–3 additional secondary electron/hole pairs.^70^ When low-energy photoelectrons are captured in the LUMO, they produce radical anion species, which can undergo decomposition reactions.^47^ Unless bond breaking reactions are exceptionally fast, they will occur only after electronic relaxation, that is, from thermally relaxed radical ions. This implies that the locations of HOMO and LUMO determine where most reactions take place. Quantum yields of tin–carbon bond breaking have recently been reported^70^ as ∼1 photon^–1^ in the range of 20–40 eV (TinOAc) and ∼3 photon^–1^ at 92 eV (TinOH). In the present case, the contribution of electrons to the photon-induced reaction via the reductive pathway (electron capture in LUMO) can be the same as in TinOH or TinOAc, but the reactions involving holes are likely to lead to decomposition of the borate anions rather than tin–carbon bond cleavage. Thus, it is not surprising that at the initial stage of the conversion, the decomposition of the tetratolylborate counterion in **2** could be observed (Figure 8). Unfortunately, for the TinPFPB **1**, similar experiments were not conclusive because of the low intensity of the characteristic C 1s → π* band at 284.7 eV and the overlap of the stronger C 1s → π* band at 287.3 eV with the transitions of the butyl groups of the tin-oxo cage.

As mentioned above, borate anions have been shown to decompose upon photochemical one-electron oxidation to triarylboron and aryl radicals. Similar pathways can be expected to occur here. The infrared spectroscopy on exposed sample **1** indicates a shift of the C–B band from 1086 to 1092 cm^–1^, indicating a change of the chemical environment of the boron [e.g., B(Ar_4_)^−^ vs BAr_3_]. The mass spectrometry analysis of 20 mJ cm^–2^ EUV-exposed material **1** indicates that the degradation products of the borate anion (fluorinated phenyl rings) readily graft onto the tin cluster. While the most abundant tin cluster species do possess one or two cleaved butyl groups, it must be noted that this is not a prerequisite for the grafting of the fluorophenyl moieties. Indeed, species of “intact” tin clusters with additional fluorophenyl rings can be observed at 2583 and 2754 Da for the mono- and bis-fluorophenylated species, respectively. The presence of these species raises the question of the structural rearrangements that the tin cluster must undergo to accommodate for the additional fluorophenyls. Unfortunately, the mass spectra do not provide structural details, and other techniques are too insensitive to reveal clear and interpretable changes at a low conversion. The coordination sphere of tin is quite flexible, and replacement of butyl groups by the aromatic radicals might be possible. Photon absorption by the tin-oxo cage can be expected to lead to the usual tin–carbon bond cleavage, which is also apparent from the XPS and IR data. This can lead to tin-centered radicals that can trap the aromatic radicals. Many reaction pathways are possible, and we have to leave the details as a challenge for future research. The addition of the fluorinated phenyl rings is sufficient to induce a solubility switch that leads to the positive tone behavior observed with using ethylbenzene or toluene as a developer in the 10–25 mJ cm^–2^ EUV dose range. The development is a complex physical-chemical process, controlled by kinetics. Possibly, a relatively small perturbation of the packing of the molecules suffices to accelerate the penetration of the solvent and the dissolution. At higher doses, the photoproducts are further degraded, probably via tin–carbon bond cleavages, to insoluble material, thereby reverting to the conventional negative tone behavior.

## 4 Conclusions

New photoresists based on the Sn_12_ cage have been synthesized, namely, TinPFPB **1** and TinTB **2**, which have bulky tetraphenylborate counteranions. TinTB **2** behaves like most Sn_12_ cage systems as a negative tone photoresist, but TinPFPB **1** can act as a positive tone resist. The difference in reactivity between the two systems is unlikely to come from the distance between two clusters, decreasing the possibility of cluster–cluster aggregation upon EUV exposure as both anions have similar sizes. The positive tone behavior is rather attributed to a combination of reactivity upon EUV irradiation of the borate as well as the ability of the pentafluorophenyl moiety to be readily grafted to the tin cage. The present paper emphasizes the importance of the study of the tin cage–anion interactions upon EUV exposure as well as a possible pathway to tune and optimize the solubility switch properties along with tuning of the Sn_12_ cage moieties.

## Data Availability

Experimental and computational data are available at https://doi.org/10.21942/uva.26210333.

## Supporting Information Available

Experimental details, crystal structure and determination data of TinPFPB, additional ^1^H, ^19^F, ^119^Sn, ^13^C NMR, MS, XAS, AFM images, and IR spectra of TinPFPB and TinPFPB thin films (PDF)
Crystal structure of TinPFPB (CIF)

## Supplementary Material

am4c08636_si_001.pdf

am4c08636_si_002.cif

## Abbreviations

PFPB: tetrakis(pentafluorophenyl)borate
TB: tetrakis(*p*-tolyl)borate
HMDS: hexamethyldisilazane
EUV: extreme ultraviolet
XPS: X-ray photoelectron spectroscopy
AFM: atomic force microscopy
XAS: X-ray absorption spectroscopy

## References

[ref1] Ewing J. J.; Brau C. A. Laser Action on the ^2^Σ^+^_1/2_→^2^Σ^+^_1/2_ Bands of KrF and XeCl. Appl. Phys. Lett. 1975, 27 (6), 350–352. 10.1063/1.88473.

[ref2] Basting D.; Djeu N.; Jain K. In Historical Review of Excimer Laser Development; Springer-Verlag, 2005; pp 8–21.

[ref3] Pol V.; Bennewitz J. H.; Escher G. C.; Feldman M.; Firtion V. A.; Jewell T. E.; Wilcomb B. E.; Clemens J. T. Excimer Laser-Based Lithography: A Deep Ultraviolet Wafer Stepper. Proc. SPIE 1986, 633, 6–16. 10.1117/12.963697.

[ref4] Ogawa T. The State-of-the-Art of ArF Excimer Laser Lithography. J. Photopolym. Sci. Technol. 1996, 9 (3), 379–386. 10.2494/photopolymer.9.379.

[ref5] Goethals A. M.; Vandenberghe G.; Pollentier I.; Ercken M.; Bisschop P. D.; Maenhoudt M.; Ronse K. Recent Progress in ArF Lithography for the 100nm Node. J. Photopolym. Sci. Technol. 2001, 14 (3), 333–340. 10.2494/photopolymer.14.333.

[ref6] De Bisschop P.; Laenens B.; Iwase K.; Yao T.; Dusa M.; Smayling M. C. Joint Optimization of Layout and Litho for SRAM and Logic Towards the 20nm Mode Using 193i. Proc. SPIE 2011, 7973, 79730B 10.1117/12.881688.

[ref7] Owa S.; Nagasaka H. Immersion Lithography: Its History, Current Status and Future Prospects. Proc. SPIE 2008, 7140, 15–1–15–12. 10.1117/12.804709.

[ref8] Matsuyama T. Exposure Tool Control for Advanced Semiconductor Lithography. Adv. Opt. Technol. 2015, 4 (4), 285–296. 10.1515/aot-2015-0026.

[ref9] Miyamoto H.; Furusato H.; Ishida K.; Tsushima H.; Kurosu A.; Tanaka H.; Ohta T.; Bushida S.; Saitou T.; Mizoguchi H. Next-Generation ArF Laser Technologies for Multiple-Patterning Immersion Lithography Supporting Leading Edge Processes. Proc. SPIE 2018, 10587, 10–1–10–8. 10.1117/12.2297316.

[ref10] Kinoshita H.; Kurihara K.; Ishii Y.; Torii Y. Soft x-ray reduction lithography using multilayer mirrors. J. Vac. Sci. Technol. B 1989, 7 (6), 1648–1651. 10.1116/1.584507.

[ref11] Lio A. EUV Photoresists: A Progress Report and Future Prospects. Synchrotron Radiat. News 2019, 32 (4), 9–14. 10.1080/08940886.2019.1634431.

[ref12] Burov A.; Pret A. V.; Gronheid R. Depth of focus in high-NA EUV lithography: a simulation study. Proc. SPIE 2022, 12293, 13 10.1117/12.2642695.

[ref13] Fujita J.; Ohnishi Y.; Ochiai Y.; Matsui S. Ultrahigh Resolution of Calixarene Negative Resist in Electron Beam Lithography. Appl. Phys. Lett. 1996, 68 (9), 1297–1299. 10.1063/1.115958.

[ref14] Hoole A. C. F.; Welland M. E.; Broers A. N. Negative PMMA as a High-Resolution Resist - the Limits and Possibilities. Semicond. Sci. Technol. 1997, 12 (9), 1166–1170. 10.1088/0268-1242/12/9/017.

[ref15] Singh V.; Satyanarayana V. S. V.; Kessler F.; Scheffer F. R.; Weibel D. E.; Sharma S. K.; Ghosh S.; Gonsalves K. E. Optimization of Processing Parameters and Metrology for Novel NCA Negative Resists for NGL. Proc. SPIE 2014, 9048, 512 10.1117/12.2045882.

[ref16] Wang Z.; Maric M. Synthesis of Narrow Molecular Weight Distribution Norbornene-Lactone Functionalized Polymers by Nitroxide-Mediated Polymerization: Candidates for 193-nm Photoresist Materials. Polymers 2014, 6 (2), 565–582. 10.3390/polym6020565.

[ref17] Carbaugh D. J.; Wright J. T.; Parthiban R.; Rahman F. Photolithography with polymethyl methacrylate (PMMA). Semicond. Sci. Technol. 2016, 31 (2), 025010 10.1088/0268-1242/31/2/025010.

[ref18] Allen R. D.; Wallraff G. M.; Hinsberg W. D.; Simpson L. L. High performance acrylic polymers for chemically amplified photoresist applications. J. Vac. Sci. Technol. B 1991, 9 (6), 3357–3361. 10.1116/1.585341.

[ref19] Ito H.; Seehof N.; Sato R.; Nakayama T.; Ueda M. Synthesis and Evaluation of Alicyclic Backbone Polymers for 193 nm Lithography. Am. Chem. Soc. 1998, 706, 208–223. 10.1021/bk-1998-0706.ch016.

[ref20] Wieberger F.; Neuber C.; Ober C. K.; Schmidt H. W. Tailored Star Block Copolymer Architecture for High Performance Chemically Amplified Resists. Adv. Mater. 2012, 24 (44), 5939–5944. 10.1002/adma.201201547. 22961836

[ref21] Novembre A.; Liu S. In Chemistry and Processing of Resists for Nanolithography; Elsevier, 2014; pp 194–286.

[ref22] Kruger S. A.; Higgins C.; Cardineau B.; Younkin T. R.; Brainard R. L. Catalytic and Autocatalytic Mechanisms of Acid Amplifiers for Use in EUV Photoresists. Chem. Mater. 2010, 22 (19), 5609–5616. 10.1021/cm101867g.

[ref23] Simone D. D.; Goethals A. M.; Roey F. V.; Zheng T.; Foubert P.; Hendrickx E.; Vandenberghe G.; Ronse K. Progresses and Challenges of EUV Lithography Materials. J. Photopolym. Sci. Technol. 2014, 27 (5), 601–610. 10.2494/photopolymer.27.601.

[ref24] Levinson H. J. Lithography in a quantum world. Jpn. J. Appl. Phys. 2023, 62 (SG), SG0802 10.35848/1347-4065/acb8be.

[ref25] Kozawa T.; Tagawa S. Radiation Chemistry in Chemically Amplified Resists. Jpn. J. Appl. Phys. 2010, 49 (3R), 030001 10.1143/JJAP.49.030001.

[ref26] Li L.; Liu X.; Pal S.; Wang S.; Ober C. K.; Giannelis E. P. Extreme Ultraviolet Resist Materials for Sub-7 nm Patterning. Chem. Soc. Rev. 2017, 46 (16), 4855–4866. 10.1039/C7CS00080D. 28650497

[ref27] Lewis S. M.; Alty H. R.; Vockenhuber M.; DeRose G. A.; Fernandez-Mato A.; Kazazis D.; Winpenny P. L.; Grindell R.; Timco G. A.; Scherer A.; Ekinci Y.; Winpenny R. E. P. Sensitivity Enhancement of a High-Resolution Negative-Tone Nonchemically Amplified Metal Organic Photoresist for Extreme Ultraviolet Lithography. J. Micro/Nanopatterning, Mater., Metrol. 2022, 21 (04), 041404 10.1117/1.JMM.21.4.041404.

[ref28] Wang X.; Tao P.; Wang Q.; Zhao R.; Liu T.; Hu Y.; Hu Z.; Wang Y.; Wang J.; Tang Y.; Xu H.; He X. Trends in photoresist materials for extreme ultraviolet lithography: A review. Mater. Today 2023, 67, 299–319. 10.1016/j.mattod.2023.05.027.

[ref29] Xu H.; Kosma V.; Giannelis E.; Ober C. K.; Sakai K. EUV photolithography: resist progress and challenges. Proc. SPIE 2018, 10583, 06–1–06–13. 10.1117/12.2302759.

[ref30] Luo C.; Xu C.; Lv L.; Li H.; Huang X.; Liu W. Review of Recent Advances in Inorganic Photoresists. RCS Adv. 2020, 10 (14), 8385–8395. 10.1039/C9RA08977B. PMC9049984 35497823

[ref31] Henke B. L.; Gullikson E. M.; Davis J. C. X-Ray Interactions: Photoabsorption, Scattering, Transmission, and Reflection at E = 50–30,000 eV, Z = 1–92. At. Data Nucl. Data Tables 1993, 54 (2), 181–342. 10.1006/adnd.1993.1013.

[ref32] Closser K. D.; Ogletree D. F.; Naulleau P.; Prendergast D. The Importance of Inner-Shell Electronic Structure for Enhancing the EUV Absorption of Photoresist Materials. J. Chem. Phys. 2017, 146 (16), 164106 10.1063/1.4981815. 28456207

[ref33] Piszczek P.; Radtke A.; Grodzicki A.; Wojtczak A.; Chojnacki J. The New Type of [Zr_6_(μ_3_-O)_4_(μ_3_-OH)_4_] Cluster Core: Crystal Structure and Spectral Characterization of [Zr_6_O_4_(OH)_4_(OOCR)_12_] (R = But, C(CH_3_)_2_Et). Polyhedron 2007, 26 (3), 679–685. 10.1016/j.poly.2006.08.025.

[ref34] Cardineau B.; Del Re R.; Marnell M.; Al-Mashat H.; Vockenhuber M.; Ekinci Y.; Sarma C.; Freedman D. A.; Brainard R. L. Photolithographic properties of tin-oxo clusters using extreme ultraviolet light (13.5nm). Microelectron. Eng. 2014, 127, 44–50. 10.1016/j.mee.2014.04.024.

[ref35] Wu L.; Baljozovic M.; Portale G.; Kazazis D.; Vockenhuber M.; Jung T.; Ekinci Y.; Castellanos S. Mechanistic Insights in Zr- and Hf-Based Molecular Hybrid EUV Photoresists. J. Micro/Nanopatterning, Mater., Metrol. 2019, 18 (01), 1 10.1117/1.jmm.18.1.013504.

[ref36] Yogesh M.; Moinuddin M. G.; Khillare L. D.; Chinthalapalli S.; Sharma S. K.; Ghosh S.; Gonsalves K. E. Organotin bearing polymeric resists for electron beam lithography. Microelectron. Eng. 2022, 260, 111795 10.1016/j.mee.2022.111795.

[ref37] Murphy M.; Upadhyay N. S.; Ali M.; Passarelli J.; Grzeskowiak J.; Weires M.; Brainard R. L. Polymerizable Olefins Groups in Antimony EUV Photoresists. J. Photopolym. Sci. Technol. 2021, 34 (1), 117–121. 10.2494/photopolymer.34.117.

[ref38] De Simone D.; Kljucar L.; Das P.; Blanc R.; Beral C.; Severi J.; Vandenbroeck N.; Foubert P.; Charley A.-l.; Oak A.; Xu D.; Gillijns W.; Mitard J.; Tokei Z.; van der Veen M.; Heylen N.; Teugels L.; Le Q. T.; Schleicher F.; Leray P.; Ronse K.; Kim I. H.; Kim I.; Park C.; Lee J.; Ryu K.; De Schepper P.; Doise J.; Kocsis M. 28nm pitch single exposure patterning readiness by metal oxide resist on 0.33NA EUV lithography. Proc. SPIE 2021, 11609, 0Q-1Q–0Q-11. 10.1117/12.2584713.

[ref39] Rohdenburg M.; Thakur N.; Cartaya R.; Castellanos S.; Swiderek P. Role of low-energy electrons in the solubility switch of Zn-based oxocluster photoresist for extreme ultraviolet lithography. Phys. Chem. Chem. Phys. 2021, 23 (31), 16646–16657. 10.1039/D1CP02334A. 34323899 PMC8359932

[ref40] Ober C.; Giannelis E. New Oxide Nanoparticle Extreme-UV Photoresists Achieve High Sensitivity; SPIE Newsroom, 2014.

[ref41] Li L.; Chakrabarty S.; Spyrou K.; Ober C. K.; Giannelis E. P. Studying the Mechanism of Hybrid Nanoparticle Photoresists: Effect of Particle Size on Photopatterning. Chem. Mater. 2015, 27 (14), 5027–5031. 10.1021/acs.chemmater.5b01506.

[ref42] Vaglio Pret A. V., Graves T., Blankenship D., Biafore J. J. Modeling and simulation of low-energy electron scattering in organic and inorganic EUV photoresists. Proc. SPIE, 2017, 10146, 09-1-09-16.

[ref43] Trikeriotis M.; Bae W. J.; Schwartz E.; Krysak M.; Lafferty N.; Xie P.; Smith B.; Zimmerman P. A.; Ober C. K.; Giannelis E. P. Development of an Inorganic Photoresist for DUV, EUV, and Electron Beam Imaging. Proc. SPIE 2010, 10146, 09–1–09–16. 10.1117/12.846672.

[ref44] Ekinci Y.; Vockenhuber M.; Hojeij M.; Wang L.; Mojarad N. M. Evaluation of EUV Resist Performance with Interference Lithography Towards 11 nm Half-Pitch and Beyond. Proc. SPIE 2013, 8679, 10–1–10–11. 10.1117/12.2011533.

[ref45] Haitjema J.; Zhang Y.; Vockenhuber M.; Kazazis D.; Ekinci Y.; Brouwer A. M. Extreme Ultraviolet Patterning of Tin-oxo Cages. J. Micro/Nanopatterning, Mater., Metrol. 2017, 16 (03), 1 10.1117/1.jmm.16.3.033510.

[ref46] Zhang Y.; Haitjema J.; Baljozovic M.; Vockenhuber M.; Kazazis D.; Jung T. A.; Ekinci Y.; Brouwer A. M. Dual-tone Application of a Tin-Oxo Cage Photoresist Under E-beam and EUV Exposure. J. Photopolym. Sci. Technol. 2018, 31 (2), 249–255. 10.2494/photopolymer.31.249.

[ref47] Bespalov I.; Zhang Y.; Haitjema J.; Tromp R. M.; van der Molen S. J.; Brouwer A. M.; Jobst J.; Castellanos S. Key Role of Very Low Energy Electrons in Tin-Based Molecular Resists for Extreme Ultraviolet Nanolithography. ACS Appl. Mater. Interfaces 2020, 12 (8), 9881–9889. 10.1021/acsami.9b19004. 32019303

[ref48] Sharps M. C.; Frederick R. T.; Javitz M. L.; Herman G. S.; Johnson D. W.; Hutchison J. E. Organotin Carboxylate Reagents for Nanopatterning: Chemical Transformations during Direct-Write Electron Beam Processes. Chem. Mater. 2019, 31 (13), 4840–4850. 10.1021/acs.chemmater.9b01440.

[ref49] Kang Y. K.; Kim H.; Lee S. J.; Oh D.-S.; Yoon Y.-H.; Kim C.-J.; Yeom G. Y.; Hwang C.-C.; Kim M.-G. Enhancement of photosensitivity and stability of Sn-12 EUV resist by integrating photoactive nitrate anion. Appl. Surf. Sci. 2024, 656, 159564 10.1016/j.apsusc.2024.159564.

[ref50] Fallica R.; Haitjema J.; Wu L.; Castellanos S.; Brouwer A. M.; Ekinci Y. Absorption Coefficient of Metal-Containing Photoresists in the Extreme Ultraviolet. J. Micro/Nanopatterning, Mater., Metrol. 2018, 17 (02), 1 10.1117/1.JMM.17.2.023505.

[ref51] Banse F.; Ribot F.; Toledano P.; Maquet J.; Sanchez C. Hydrolysis of Monobutyltin Trialkoxides: Synthesis and Characterizations of {(BuSn)_12_O_14_(OH)_6_}(OH)_2_. Inorg. Chem. 1995, 34 (25), 6371–6379. 10.1021/ic00129a023.

[ref52] Eychenne-Baron C.; Ribot F.; Sanchez C. New Synthesis of the Nanobuilding Block {(BuSn)_12_O_14_(OH)_6_}^2+^ and Exchange Properties of {(BuSn)_12_O_14_(OH)_6_}(O_3_SC_6_H_4_CH_3_)_2_. J. Organomet. Chem. 1998, 567 (1–2), 137–142. 10.1016/S0022-328X(98)00676-7.

[ref53] Eychenne-Baron C.; Ribot F.; Steunou N.; Sanchez C.; Fayon F.; Biesemans M.; Martins J. C.; Willem R. Reaction of Butyltin Hydroxide Oxide with p-Toluenesulfonic Acid: Synthesis, X-ray Crystal Analysis, and Multinuclear NMR Characterization of {(BuSn)_12_O_14_(OH)_6_}(4-CH_3_C_6_H_4_SO_3_)_2_. Organometallics 2000, 19 (10), 1940–1949. 10.1021/om990877a.

[ref54] Haitjema J.; Wu L.; Giuliani A.; Nahon L.; Castellanos S.; Brouwer A. M. UV and VUV-Induced Fragmentation of Tin-Oxo Cage Ions. Phys. Chem. Chem. Phys. 2021, 23 (37), 20909–20918. 10.1039/D1CP03148A. 34533559

[ref55] Sadegh N.; Evrard Q.; Kraus P. M.; Brouwer A. M. XUV Absorption Spectroscopy and Photoconversion of a Tin-Oxo Cage Photoresist. J. Phys. Chem. C 2024, 128 (9), 3965–3974. 10.1021/acs.jpcc.3c07480. PMC10926160 38476827

[ref56] Ma J. H.; Needham C.; Wang H.; Neureuther A.; Prendergast D.; Naulleau P. Mechanistic Advantages of Organotin Molecular EUV Photoresists. ACS Appl. Mater. Interfaces 2022, 14 (4), 5514–5524. 10.1021/acsami.1c12411. 35073690

[ref57] Williams J. L. R.; Doty J. C.; Grisdale P. J.; Searle R.; Regan T. H.; Happ G. P.; Maier D. P. Boron Photochemistry. I. Irradiation of Sodium Tetraarylborates in Aqueous Solution. J. Am. Chem. Soc. 1967, 89 (20), 5153–5157. 10.1021/ja00996a013.

[ref58] Grisdale P. J.; Williams J. L. R.; Glogowski M. E.; Babb B. E. Boron Photochemistry. Possible Role of Bridged Intermediates in the Photolysis of Borate Complexes. J. Org. Chem. 1971, 36 (4), 544–549. 10.1021/jo00803a012.

[ref59] Hewavitharanage P.; Danilov E. O.; Neckers D. C. Pentafluorophenyl Transfer: A New Group-Transfer Reaction in Organoborate Salts. J. Org. Chem. 2005, 70 (26), 10653–10659. 10.1021/jo050695s. 16355982

[ref60] Murphy S. T.; Zou C.; Miers J. B.; Ballew R. M.; Dlott D. D.; Schuster G. B. Tetraarylborates {[Ar]_4_B^–^}: Estimation of Oxidation Potentials and Reorganization Energies from Electron-Transfer Rates. J. Phys. Chem. 1993, 97 (50), 13152–13157. 10.1021/j100152a020.

[ref61] Haitjema J.; Castellanos S.; Lugier O.; Bespalov I.; Lindblad R.; Timm M.; Bülow C.; Zamudio-Bayer V.; Lau J. T.; von Issendorff B.; Hoekstra R.; Witte K.; Watts B.; Schlathölter T.; Brouwer A. M. Soft X-ray Absorption and Fragmentation of Tin-Oxo Cage Photoresists. Phys. Chem. Chem. Phys. 2024, 26 (7), 5986–5998. 10.1039/D3CP05428D. 38293812

[ref62] Hair M. L.; Hertl W. Reaction of Hexamethyldisilazane with Silica. J. Phys. Chem. 1971, 75 (14), 2181–2185. 10.1021/j100683a020.

[ref63] Tsyganenko A. A.; Filimonov V. N. Infrared Spectra of Surface Hydroxyl Groups and Crystalline Structure of Oxides. J. Mol. Struct. 1973, 19, 579–589. 10.1016/0022-2860(73)85136-1.

[ref64] Nannarone S.; Borgatti F.; DeLuisa A.; Doyle B. P.; Gazzadi G. C.; Giglia A.; Finetti P.; Mahne N.; Pasquli L.; Pedio M.; Selvaggi G.; Naletto G.; Pelizzo M. G.; Tondello G. The BEAR Beamline at Elettra. AIP Conf. Proc. 2004, 705, 450–453. 10.1063/1.1757831.

[ref65] Zhang Y.; Haitjema J.; Liu X.; Johansson F.; Lindblad A.; Castellanos S.; Ottosson N.; Brouwer A. M. Photochemical Conversion of Tin-Oxo Cage Compounds Studied Using Hard X-ray Photoelectron Spectroscopy. J. Micro/Nanopatterning, Mater., Metrol. 2017, 16 (2), 023510 10.1117/1.JMM.16.2.023510.

[ref66] Norman P.; Dreuw A. Simulating X-ray Spectroscopies and Calculating Core-Excited States of Molecules. Chem. Rev. 2018, 118 (15), 7208–7248. 10.1021/acs.chemrev.8b00156. 29894157

[ref67] Triguero L.; Pettersson L. G. M.; Ågren H. Calculations of Near-Edge X-ray-Absorption Spectra of Gas-Phase and Chemisorbed Molecules by Means of Density-Functional and Transition-Potential Theory. Phys. Rev. B: Condens. Matter Mater. Phys. 1998, 58 (12), 8097–8110. 10.1103/PhysRevB.58.8097.

[ref68] te Velde G.; Bickelhaupt F. M.; Baerends E. J.; Guerra C. F.; van Gisbergen S. J. A.; Snijders J. G.; Ziegler T. Chemistry with ADF. J. Comput. Chem. 2001, 22 (9), 931–967. 10.1002/jcc.1056.

[ref69] Haitjema J.; Wu L.; Giuliani A.; Nahon L.; Castellanos S.; Brouwer A. M. Photo-induced Fragmentation of a Tin-oxo Cage Compound. J. Photopolym. Sci. Technol. 2018, 31 (2), 243–247. 10.2494/photopolymer.31.243.

[ref70] Sadegh N.; Evrard Q.; Mahne N.; Giglia A.; Nannarone S.; Brouwer A. M. Electron Generation in Tin-oxo Cage Extreme Ultraviolet Photoresists. J. Photopolym. Sci. Technol. 2023, 36 (5), 373–378. 10.2494/photopolymer.36.373.

