# Supplementary material for: Extreme Ultraviolet Photoresponse of Organotin-Based Photoresists with Borate Counteranions
Source: ACS Appl Mater Interfaces. 2024 Aug 5;16(32):42947–56. doi: 10.1021/acsami.4c08636 (PMC11331440; doi:10.1021/acsami.4c08636)
Supplement: Supplementary file 1 — am4c08636_si_001.pdf [file am4c08636_si_001.pdf]

Supporting Information

Extreme Ultraviolet Photoresponse of Organotin-based Photoresists with Borate Counter Anions

Quentin Evrard,[a,b]* Najmeh Sadegh,*[a] *Simon Mathew,*[b] *Ed Zuidinga,*[b] *Benjamin Watts,*[c] *Maximilian Paradiz Dominguez,*[b] *Angelo Giglia,*[d] *Nicola Mahne,*[d] *Stefano Nannarone,*[d] *Akira Nishimura,*[e] *Tsuyoshi Goya,*[e] *Takuo Sugioka,*[e] *Michaela Vockenhuber,*[c] *Yasin Ekinci,*[c] *Albert M. Brouwer*[a,b]*

[a]Advanced Research Center for Nanolithography ARCNL, Science Park 106, 1098 XG Amsterdam, The Netherlands

[b]van't Hoff Institute for Molecular Sciences, University of Amsterdam, P.O. Box 94157, 1090 GD Amsterdam, The Netherlands

[c]Paul Scherrer Institute, Forschungsstrasse 111, 5232 Villigen PSI, Switzerland

[d]CNR-IOM, Strada Statale 14 km 163,5 - 34149 Basovizza – Trieste, Italy

[e]Nippon Shokubai, 5-8 Nishi Otabi-cho, Suita, Osaka, 564-0034, Japan

Corresponding Author

*Quentin Evrard, Van't Hoff Institute for Molecular Sciences, University of Amsterdam, P.O. Box 94157, 1090 GD Amsterdam, The Netherlands; Email: q.j.o.evrard@uva.nl

*Albert M. Brouwer, Van't Hoff Institute for Molecular Sciences, University of Amsterdam, P.O. Box 94157, 1090 GD Amsterdam, The Netherlands; Email: A.M.Brouwer@uva.nl

Table of contents

1. Synthesis and characterization S4
2. X-ray diffraction S6
3. Thin film formation S8
4. Atomic Force Microscopy S8
5. X-Ray Photoemission Spectroscopy S9
6. EUV exposures S9
7. X-ray absorption spectroscopy S10
7.1 XAS data acquisition S10
7.2 XAS Data Processing S11
7.3 XAS computational data S13
7.4 XAS calculations using TP method S14
8. Mass spectrometry S15
9. Infrared spectroscopy S15
10. Figures S16
11. References S28
Figure S1: XAS of **1** S12
Figure S2: XAS of **2** S12
Figure S3: XAS of **4** S13
Figure S4: Visual representation of LUMO (A), HOMO (B) and highest σ (C) of **2**. S14
Figure S5: Mass spectra of negative ions of TinPFPB **1** obtained via ESI. S16
Figure S6: Mass spectra of positive ions of TinPFPB **1** obtained via ESI. S16
Figure S7: Mass spectra of positive ions of TinPFPB **1** film exposed to 20 mJ/cm^2 of EUV. S17
Figure S8: Selected part of mass spectra of TinPFPB **1** film exposed to 20 mJ/cm^2 of EUV S17
Figure S9: Selected part of mass spectra of TinPFPB **1** film exposed to 20 mJ/cm^2 of EUV S18
Figure S10: ^{19}F NMR spectra of bulk TinPFPB **1** in MeOD. S18
Figure S11: Selected area of ^{19}F NMR spectra of bulk TinPFPB **1** in MeOD. S19
Figure S12: ^{1}H NMR spectra of bulk TinPFPB **1** in MeOD. S19
Figure S13: Selected areas of ^{1}H NMR spectra of bulk TinPFPB **1** in MeOD. S20
Figure S14: Selected area of ^{1}H NMR spectra of bulk TinPFPB **1** in $CDCl_3$. S20
Figure S15: ^{119}Sn {^{1}H} NMR spectra of bulk TinPFPB **1** in MeOD. S21
Figure S16: ^{13}C NMR spectra of bulk TinPFPB **1** in MeOD. S21
Figure S17: ^{11}B NMR spectra of bulk TinPFPB **1** in MeOD. S22
Figure S18: ^{119}Sn {^{1}H} NMR spectra of bulk TinTB **2** in MeOD. S22
Figure S19: ^{1}H NMR spectra of bulk TinTB **2** in MeOD. S23

Figure S20: Selected areas of ^{1}H NMR spectra of bulk TinTB **2** in MeOD. S23
Figure S21: ^{13}C NMR spectra of bulk TinTB **2** in MeOD..S24
Figure S22: ^{11}B NMR spectra of bulk TinTB **2** in MeOD..S24
Figure S23: IR spectra of bulk (ATR) and thin film (reflectance) of TinPFPB **1**. S25
Figure S24: IR spectra of film of TinPFPB **1** exposed to EUV..S25
Figure S25: ^{1}H NMR of 1 in MeOD (blue/green) and $CDCl_3$ (brown)..S26
Figure S26: X-ray photoelectron spectra of C1s edge of TinPFPB **1** after in situ 92 eV exposure. ...S26
Figure S27: XAS at the F K-edge of 1 (blue) and 4 (red). S27
Figure S28: AFM image of TinPFPB (top) and TinTB (bottom) S29
Table S1: Crystal structure and determination data of TinPFPB **1.**...S7
Table S2: Orbital energies (eV) of highest occupied and lowest unoccupied π, σ, σ* and π* MO's..S13

1. Synthesis and characterization

The acid form of $B(PFP)_4^-$ is obtained from $NaB(PFP)_4$ by reaction with a slight excess (1.07:1) of aqueous HCl (37%) in water at 40°C for 30 minutes. The product is extracted with diethyl ether five times while the aqueous phase is discarded. The organic phase is evaporated via heating at 35°C. The obtained viscous liquid is dispersed in water and sonicated for two hours. The dispersion is then cooled to room temperature, filtered and washed with water three times. The resulting white powder is used without further purification (yield 60–70%).

The synthesis of the Sn_{12} oxo hydroxo cage with hydroxide anions (TinOH, **3**) is performed as in previous work[1] via the hydrolysis of butyltin-hydroxide-oxide in the presence of p-toluene-sulfonic acid followed by exchange of the toluene-sulfonate for hydroxide counterions using tetramethylammonium hydroxide aqueous (25%) solution. After recrystallization from isopropanol/water (10:1), TinOH (250 mg; 0.10 mmol) is dispersed in 4 mL of toluene and mixed with a 10 mL aqueous solution of tetrakis(pentafluorophenyl)boric acid (136 mg; 0.20 mmol). After 2 hours of vigorous stirring and ultrasonication, the resulting two-phase system is allowed to cool down for two additional hours. The toluene layer is separated, filtered and washed with distilled water (5 mL) three times. The white powder resulting after evaporation of the solvent is used as photoresist material without further purification (yield of **TinPFPB 1**: 317 mg, 83%) ^{1}H NMR (400 MHz, $CDCl_3$): δ = 1.73 (m, Sn_5-C**H_2**-C**H_2**-CH_2-CH_3), 1.44 (m, Sn_5-CH_2-CH_2-C**H_2**-CH_3, Sn_6-CH_2-C**H_2**-CH_2-CH_3), 1.28 (m, Sn_6-CH_2-CH_2-C**H_2**-CH_3, J = 7.2 Hz), 1.14 (m, Sn_6-C**H_2**-CH_2-CH_2-CH_3), 0.97 (t, Sn_5-/-C**H_3** J = 7.3 Hz), 0.85 (t, Sn_6-/-C**H_3** J = 7.3 Hz). ^{11}B NMR (128.37 MHz, MeOD): δ = -16.74; ^{13}C NMR (100.62 MHz, MeOD): δ = 29.02, 28.17, 27.67, 27.35, 27.26, 22.55, 14.08, 13.94; ^{19}F NMR (282.35 MHz, MeOD): δ = -133.73, -165.69

(t, J = 17 Hz), -169.50 (t, J = 20 Hz); $^{119}Sn\{^1H\}$ NMR (149.15 MHz, MeOD): δ = -284.92, -475.98; (Sn_5 refers to the 6 tin atoms that are 5-coordinated in the tin-oxo-hydroxo cage while Sn_6 refers to the 6 tin atoms that are 6-coordinated, see Scheme 1 in main text). IR (ATR, cm^{-1})): 3636 (υ(OH)); 2953 ($\upsilon_{as(}$$CH_3$)); 2920 ($\upsilon_{as}$($CH_2$)); 2870 ($\upsilon_s$($CH_3$)); 2855 ($\upsilon_s$($CH_2$)); 1641,1515, 1458 (C=C ring); 1270 (υ(CF)), 1085 (υ(B-C)); 979; 710 (def(C-F));

TinTB 2 is synthesised from TinOH (250 mg; 0.10 mmol) dispersed in 6 mL of toluene and a 10 mL aqueous dispersion of tetrakis(*p*-tolyl)boric acid (75.2 mg; 0.20 mmol). After two hours of vigorous stirring and ultrasonication the solvent is evaporated and the off-white solid is collected. (123 mg, 38%) ^1H NMR (300 MHz, MeOD): δ = 7.13-7.19 (m, B-[**o-H**Ph-CH_3]$_4$), 6.79 (d, B-[**m-H**Ph-CH_3]$_4$ J = 7.4 Hz), 2.20 (s, B-[Ph-C$\mathbf{H_3}$]$_4$), 1.71 (m, Sn_5-CH_2-C$\mathbf{H_2}$-CH_2-CH_3, J = 7.3 Hz), 1.39-1.58 (m, Sn_6-CH_2-C$\mathbf{H_2}$-CH_2-CH_3 and Sn_5-C$\mathbf{H_2}$-CH_2-C$\mathbf{H_2}$-CH_3), 1.32 (m, Sn_6-CH_2-CH_2-C$\mathbf{H_2}$-CH_3 J = 7.1 Hz), 1.03 (m, Sn_6-C$\mathbf{H_2}$-CH_2-CH_2-CH_3) 0.96 (t, Sn_5-/-C$\mathbf{H_3}$ J = 7.1 Hz), 0.89 (t, Sn_6-/-C$\mathbf{H_3}$ J = 7 Hz).^{11}B NMR (128.37 MHz, MeOD): δ = -7.30; ^{13}C NMR (100.62 MHz, MeOD): δ = 135.85, 129.62, 125.69, 28.00, 26.95, 26.69, 26.01, 23.86, 20.74, 19.88, 12.81, 12.74; $^{119}Sn\{^1H\}$ NMR (149.15 MHz, MeOD): δ = -286.79, -458.61.

The NMR spectra are shown in Figures S7–S13.

Single crystals of TinPFPB **1** were obtained from the powder obtained above and dissolving it in a 90°C 50/50 mixture of toluene/trifluorotoluene followed by a slow cooling to initiate nucleation then slow evaporation was used to increase the crystals size.

2. X-ray diffraction

Single-crystal X-ray diffraction X-ray diffraction data of TinPFPB were measured on a Bruker D8 Quest Eco diffractometer using graphite monochromated (Triumph) Mo K_α radiation (λ = 0.71073 Å) and CPAD Photon III C14 detector. The sample was cooled with N_2 to 100 K with a Cryostream 700 (Oxford Cryosystems). Intensity data were integrated using the SAINT software.[2] Absorption correction and scaling was executed with SADABS.[3] The structures were solved using intrinsic phasing with the program SHELXT 2018/2.[4] Least-squares refinement was performed with SHELXL-2018/3.[5] All non-hydrogen atoms were refined with anisotropic displacement parameters. The hydrogen atoms were introduced at calculated positions with a riding model. The resulting CIF files reveal no A-level alerts. The crystal data are summarized in Table S1. The X-ray crystallographic data were deposited at the Cambridge Crystallographic Data Centre (CCDC) under the deposition number CCDC 2332946. These data can be obtained free of charge from The Cambridge Crystallographic Data Centre via www.ccdc.cam.ac.uk/structures

Table S1: Crystal structure and determination data of TinPFPB **1.**

CCDC number	2332946
Empirical formula	$C_{96}H_{114}B_2F_{40}O_{20}Sn_{12}$
Formula weight	3793.77
Temperature [K]	100(2)
Crystal system	monoclinic
Space group (number)	$P\,2_1/n$(14)
a [Å]	15.1358(12)
b [Å]	14.5032(12)
c [Å]	27.104(2)
α [°]	90
β [°]	96.323(4)
γ [°]	90
Volume [Å^3]	5913.6(8)
Z	2
ρ_{calc} [gcm^{-3}]	2.131
μ [mm^{-1}]	2.612
F(000)	3640
Crystal size [mm^3]	0.135×0.124×0.105
Crystal colour	colourless
Crystal shape	block
Radiation	MoK_α (λ=0.71073 Å)
2Θ range [°]	4.29 to 64.14 (0.67 Å)
Index ranges	-22 ≤ h ≤ 22<br>-21 ≤ k ≤ 21<br>-40 ≤ l ≤ 40
Reflections collected	489620
Independent reflections	20597 R_{int} = 0.0869, R_{sigma} = 0.0365
Completeness to Θ = 25.242°	99.9 %
Data / Restraints / Parameters	20597/6/827
Goodness-of-fit on F^2	1.328
Final R indexes [I≥2σ(I)]	R_1 = 0.0669, wR_2 = 0.1065
Final R indexes [all data]	R_1 = 0.0801, wR_2 = 0.1099
Largest peak/hole [eÅ^{-3}]	1.44/-1.85

3. Thin film formation

A 10 mg.mL^{-1} solution of TinPFPB **1** is prepared in trifluorotoluene and sonicated for 10 minutes. For TinTB **2** a 10 mg.mL^{-1} solution in fluorobenzene was prepared and sonicated for 10 minutes. The solution of the resist of interest is then filtered using 0.2 μm PTFE filter and spincoated directly. The substrates used for EUV lithography were silicon wafers (Siegert Wafer, CZ growth, p-type B-doped, <100>, resistivity 5–10 Ohm-cm) used without additional cleaning. The samples for X-ray photoelectron spectroscopy are prepared from the same wafers diced in 25 × 25 mm substrates previously cleaned with a base piranha solution heated to 80°C for 15 minutes, rinsed with isopropanol and dried with nitrogen flow. The substrates are then cleaned using an low-pressure oxygen plasma cleaner (Diener Electronic Pico GR-200-PCCE) with a two-minute oxygen plasma with a 0.2 mbar working pressure. The substrates are then sputter coated with a 5 nm chromium adhesion layer followed by 30 nm of gold (Leica EM ACE600 double sputter coater). The thin films were obtained via spin coating at 2000 rpm with a 750 rpm/s acceleration and 45 second spinning time, no post-application or post-exposure baking are performed. The thickness of the obtained films using this procedure is within the 30–35 nm range and their roughness determined by AFM is 0.7 nm RMS regardless of the substrate used.

4. Atomic Force Microscopy

Atomic Force Microscopy for contrast curve measurement was performed on a Bruker Dimension Icon using tapping mode with Bruker RTESPA tips (150 kHz, 5 N.m^{-1}). Fields of 25 × 5 μm were measured with 256 samples per line and a scanning frequency of 0.3 Hz. Line space pattern is performed using tapping mode with Bruker TESP-SS tips (320 kHz, 42 N/m), fields of 3 × 0.6 μm were measured with 256 samples per line and a scanning frequency of 0.6 Hz. Raw

data were corrected using the truncated mean method and flattened by mean plane. No further corrections were made.

5. X-Ray Photoemission Spectroscopy

Samples prepared as described above were exposed to EUV and measured in-situ at the IOM-CNR BEAR beamline[6] [https://www.elettra.eu/elettra-beamlines/bear.html] (Elettra synchrotron, Trieste, Italy) using a hemispherical deflection electron analyzer (66 mm mean radius) driven at constant pass energy. Spectra were collected at normal emission with the beam hitting the sample at 45° with respect to the surface normal angle. The sample is first exposed to 92 eV (EUV) irradiation at the desired dose, then XPS is performed with an incident energy of 400 eV for the carbon 1s photoelectron peak with triplicate measurements on freshly exposed spots to increase the signal-to-noise ratio. The calibration in energy of the obtained spectra was made according to previously obtained data.[7] Because of the radiation sensitivity of the material, extra care has been taken to minimize the spectra acquisition time to remain below 3 minutes per scan, the effect of the measurement scan was checked by performing three measurements on the same spot of the sample in a row and comparing the obtained spectra, which showed negligible changes.

6. EUV exposures

The EUV exposures for the contrast curves and IR spectroscopy were performed at the XIL-II Beamline[8] (Paul Scherrer Institute, Switzerland) on samples prepared as described in section 3 above. The samples were exposed via an open frame non-contact mask of 0.5 × 0.5 mm square and the dose of the exposed area was controlled via the beam shutter time. For the samples for mass spectrometry, the open frame non-contact mask was replaced with a 1 × 1 mm square and multiple areas of the sample were exposed at the same dose over an area of 12 × 12 mm.

7. X-ray absorption spectroscopy

7.1 XAS data acquisition

Measurements were made using the Scanning Transmission X-ray Microscope at the PolLux beamline[9] of the Paul Scherrer Institute, as described previously.[10, 11] Samples were spin coated on SiN windows (30 nm thickness) on a silicon support. For recording spectra, samples of 5 × 5 mm were used, with 25 windows of 150 × 150 μm. By using relatively high concentrations of substrates we could obtain thicker films, with higher absorbance than in our previous work.[10] One of the windows was punctured to obtain a reference channel. For each energy, transmission of sample and of reference were measured by scanning a line of 25 μm in the center of a window, where the film is thicker than at the edges. Because moving the sample takes more time than changing the photon energy, this approach is more time consuming than measuring the whole spectrum for the sample and then for the reference. However, by minimizing the time difference between sample and reference measurement at each energy, the slow variations in the X-ray fluence were better characterized for a more accurate intensity normalization and resulting in more reproducible spectra.

For EUV irradiations, samples of 7.5 × 7.5 mm were used with SiN windows of 3 × 3 mm. Nine squares of 500 × 500 μm were irradiated with 92 eV photons at the XIL-II beamline.[8] To identify the squares, the one in the center was displaced by 100 μm from the middle of the 3 × 3 array. Difference spectra were measured by scanning a sample and a reference line on an exposed and an unexposed area, respectively, as close together as possible.

7.2 XAS Data Processing

Absorbance $A(E)$ is calculated from transmission spectra measured through the sample channel I_s and the reference channel I_r as $A = \ln(I_r / I_s)$. The reference was a hole in the sample and SiN membrane. The spectrum of the SiN membrane was measured separately and subtracted from $A(E)$. To obtain the spectrum that is attributable to C1s absorption, the pre-edge absorption in the range 270–280 eV was fitted to a straight line and subtracted from $A(E)$ over the whole range of the measurement (270–350 eV).

To obtain a smoother representation of the data for graphical comparison of the different species (Figure 7A), the absorption spectra were fitted to a sum of gaussians and a step function. These fits are shown in figures S1–3. Finally, for comparison of different measurements, which used samples that have different and not precisely known film thicknesses, the calculated cross section[12] at 340 eV (σ_{340} = 0.6808 Mb (1 megabarn = 10^{-28} m^2) per C atom) was used: the fitted absorption was multiplied by $n_C\sigma_{340}$ (n_C = number of C atoms) and divided by the experimental absorption at 340 eV. Thus, the spectra shown in Figure 7A in the main text represents cross sections scaled to the calculated cross sections for C1s photoionization. We consider this a meaningful procedure because at 340 eV no specific transitions are found and only photoionization occurs at that energy.

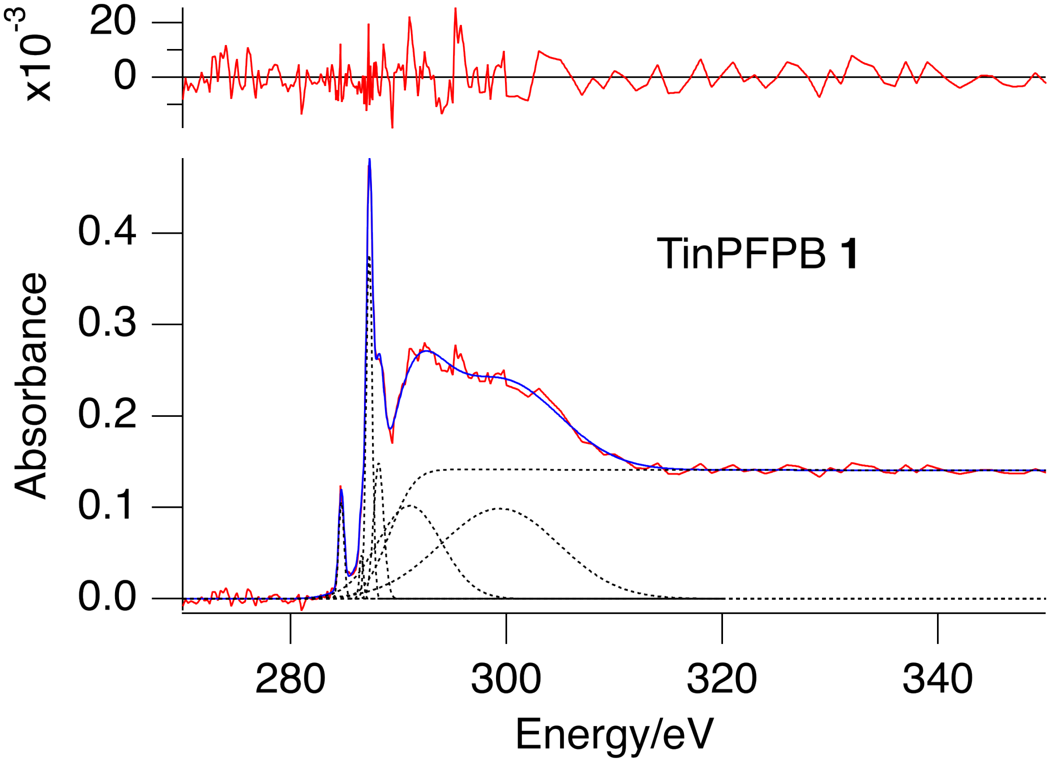

Figure S1: XAS of **1**, red is experimental data, blue is fitting and the residual fit is plotted on top.

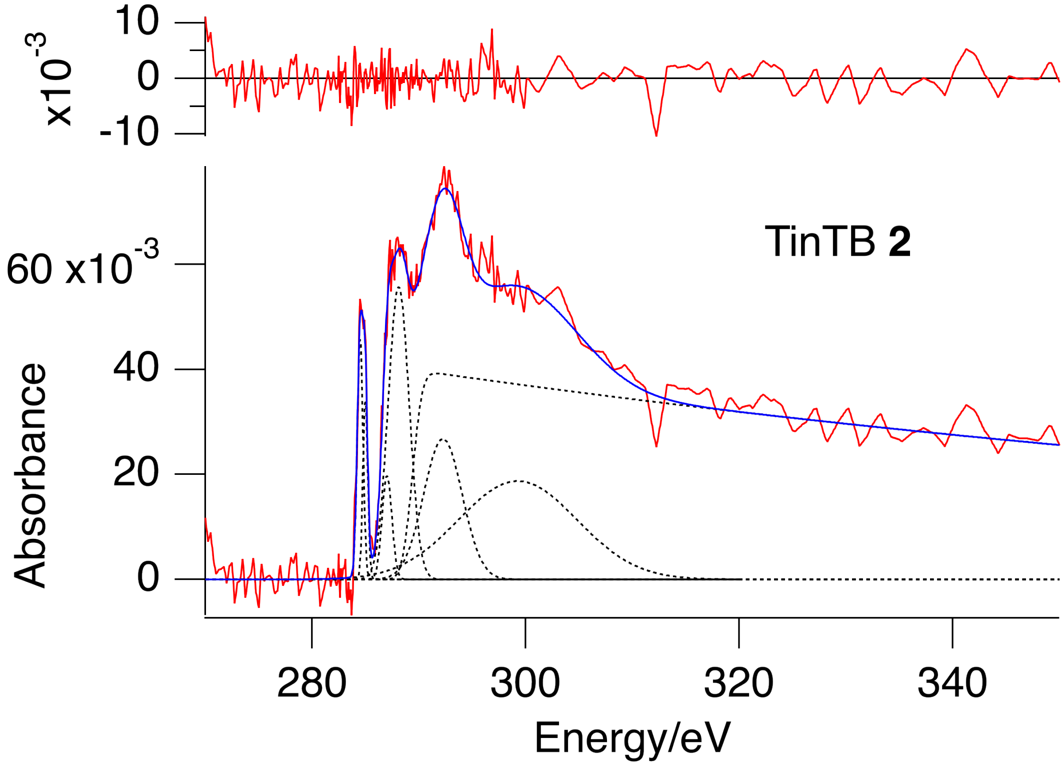

Figure S2: XAS of **2**, red is experimental data, blue is fitting and the residual fit is plotted on top.

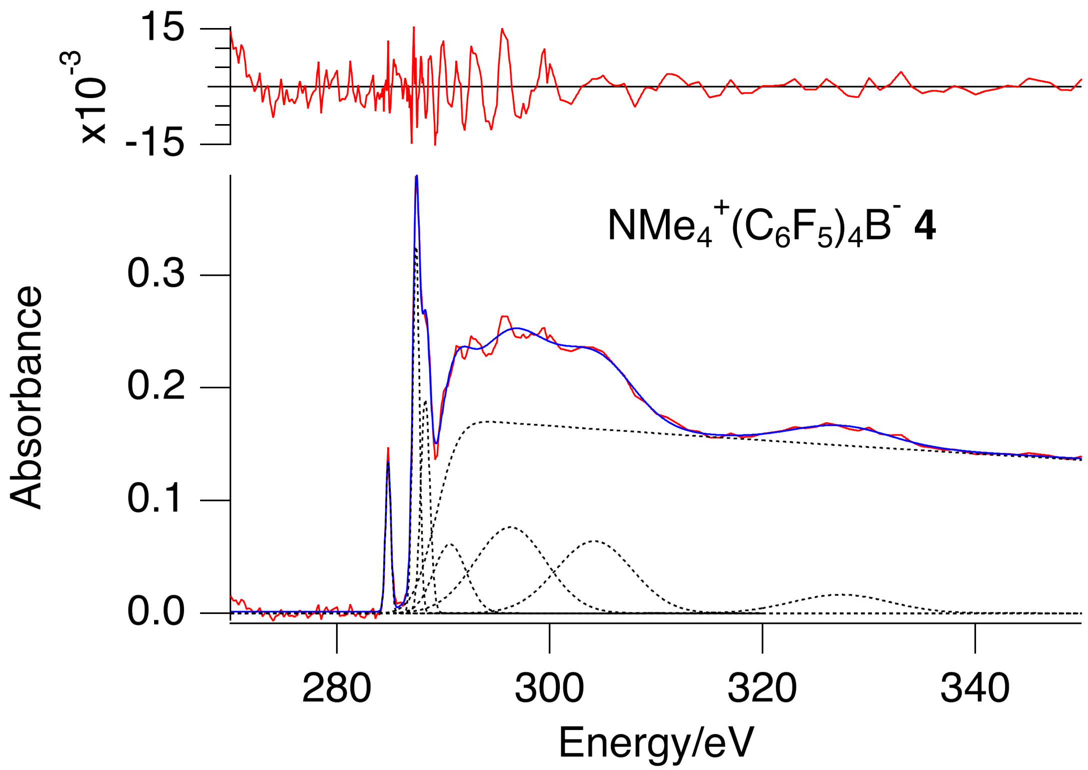

Figure S3: XAS of **4**, red is experimental data, blue is fitting and the residual fit is plotted on top.

7.3 XAS computational data

Geometries of tin-oxo cages with counterions were optimized at the B3LYP/LANL2DZ level using Gaussian16.[13]

Table S2: Orbital energies (eV) of highest occupied and lowest unoccupied π, σ, σ* and π* MO's.

compound	HOMO(π)	HOMO(σ)	LUMO(σ*)	LUMO(π*)
**1**	-7.26	-8.16	-2.61	-1.69
**2**	-4.93	-7.93	-2.03	-0.04
**3**		-6.36	-1.01	

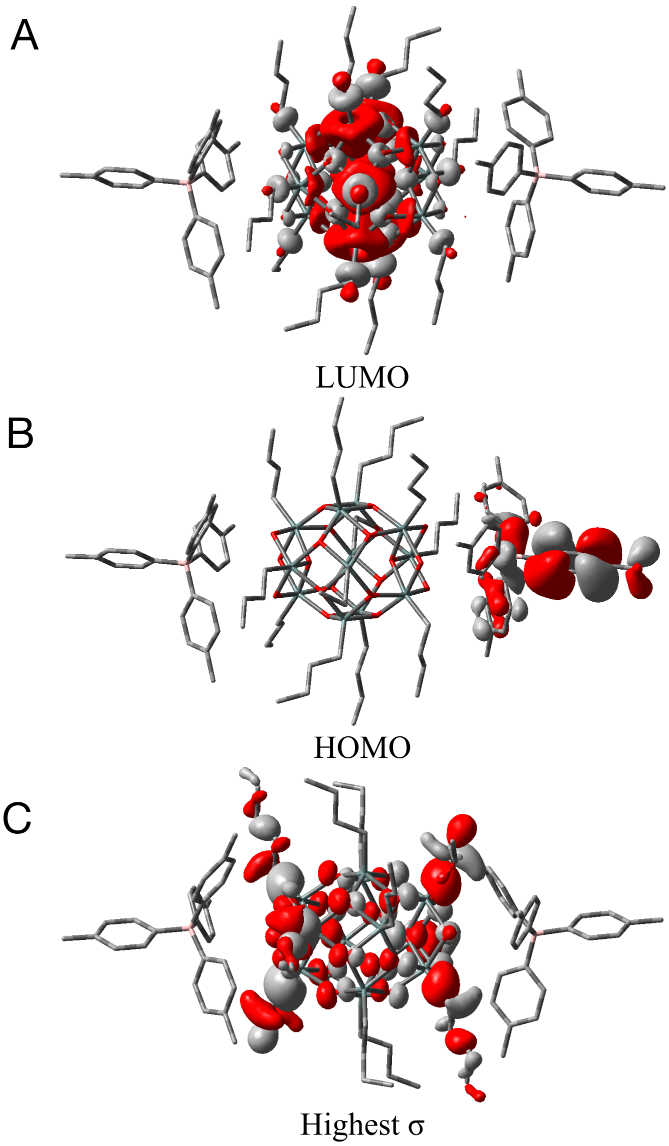

Figure S4: Visual representation of LUMO (A), HOMO (B) and highest-energy σ MO (C) of **2**.

7.4 XAS calculations using TP method[10, 11]

The structures of the borate anions were optimized using AMS2023[14] at the PBE-D3(BJ)/TZP level using the full set of core orbitals. Transition potential calculations were carried out using the LDA functional for each of the C atoms of one of the aromatic rings. The ring atoms had an AUG/ATZP large core basis set that does not explicitly include the 1s basis functions. On the

carbon atom of interest, a full AUG/ATZ2P basis set was used. The 1s level of this atom was filled with 1.5 electrons. For the atoms of the other three rings a TZ2P/large-core basis set was used.

8. Mass spectrometry

TinPFPB **1** powder was dissolved in ethylbenzene and directly measured via electrospray ionization using a JEOL, AccuTOF LC-Plus (JMS-T100LP). For the samples of exposed TinPFPB **1** films, the samples were prepared as described above and then exposed to EUV at the Paul Scherrer Institute on the XIL-II beamline (flood exposure area of 12 × 12 mm), dissolved using 1 mL of ethylbenzene and then measured using electrospray in the same conditions as for the bulk sample.

9. Infrared spectroscopy

Samples were prepared on gold substrates (SI section 3) and then irradiated at the XIL-II beamline (SLS-Paul Scherrer Institute, Switzerland) on 500 µm wide squares at different EUV doses. Each spot on the sample was then measured in reflectance mode using a Perkin Elmer Spotlight 200i FT-IR infrared microscope in a nitrogen atmosphere to mitigate CO_2/H_2O absorption with an accumulation of 128 spectra. The obtained spectra are then background corrected using multiple points to allow proper stacking and comparison of the spectra.

10. Figures

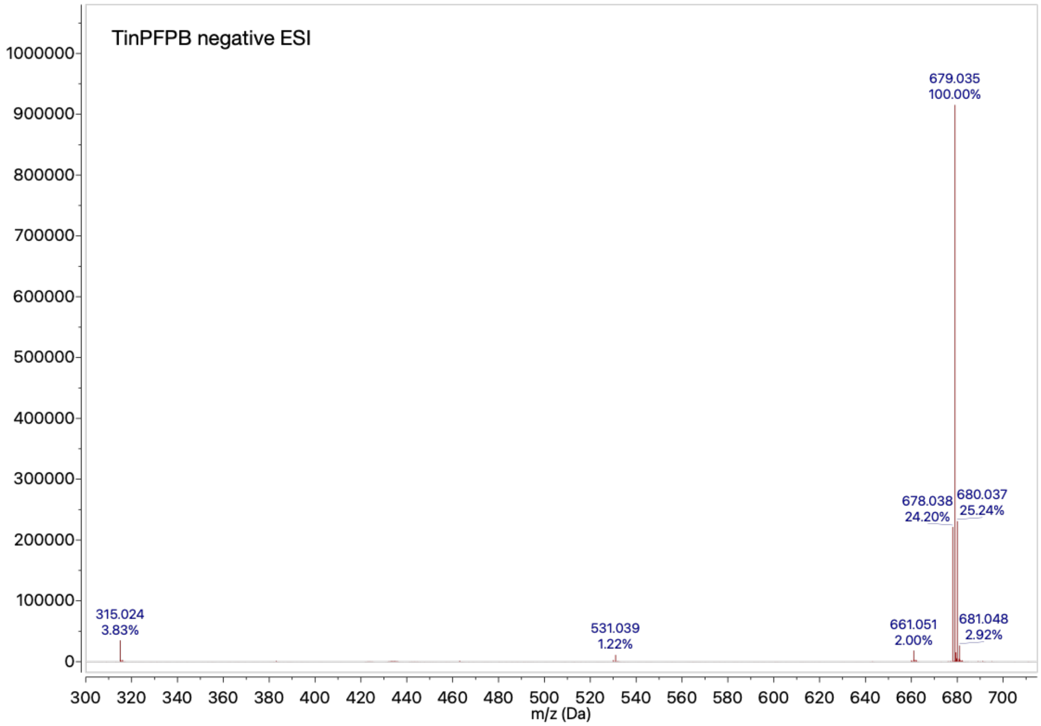

Figure S5: Mass spectra of negative ions of TinPFPB **1** obtained via Electron Spray Ionization in an ethylbenzene solution.

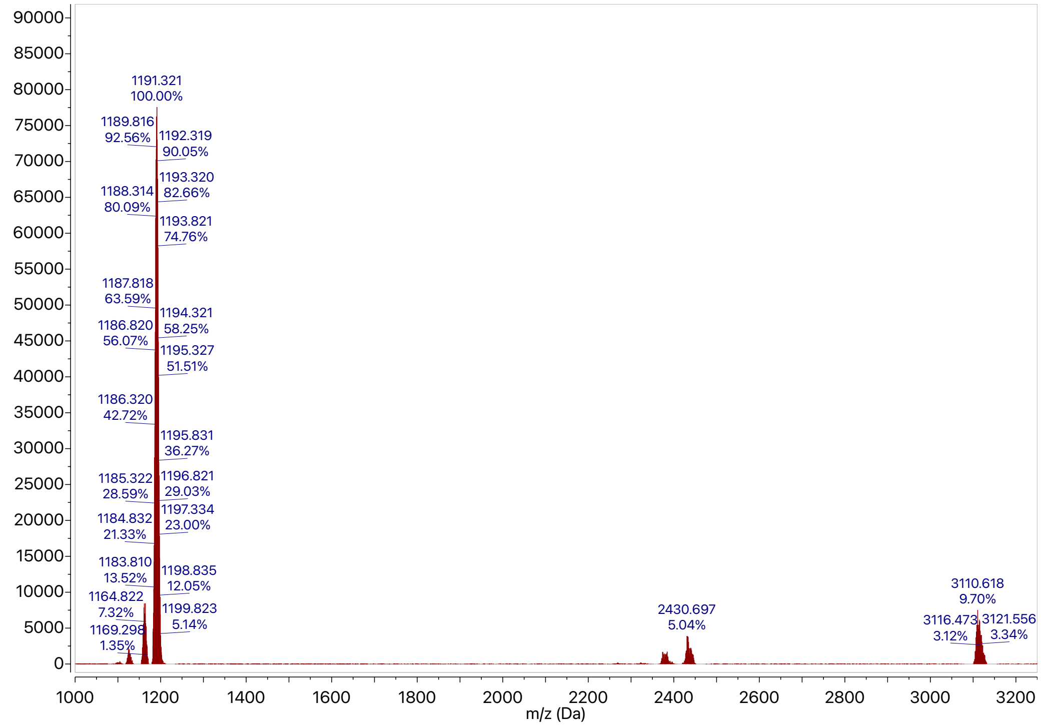

Figure S6: Mass spectra of positive ions of TinPFPB **1** obtained via Electron Spray Ionization in an ethylbenzene solution.

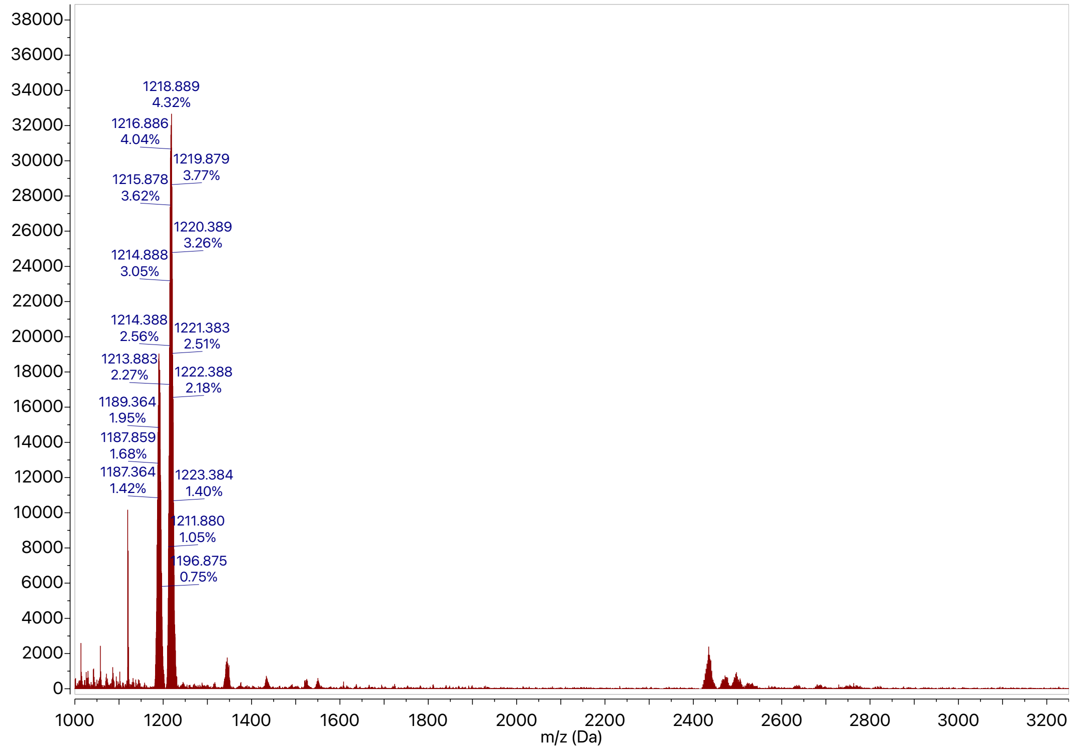

Figure S7: Mass spectra of positive ions of TinPFPB **1** film exposed to 20 mJ/cm^2 of EUV and developed with ethylbenzene obtained via Electron Spray Ionization.

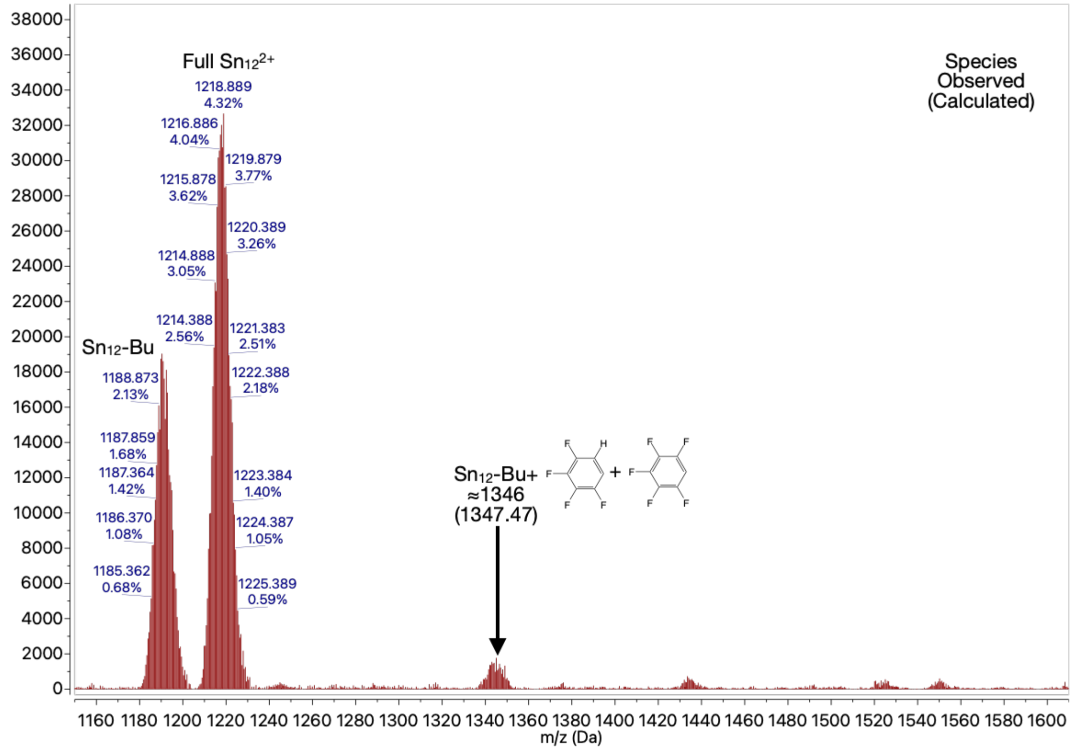

Figure S8: Selected part of mass spectra of positive ions of TinPFPB **1** film exposed to 20 mJ.cm^{-2} of EUV and developed with ethylbenzene obtained via Electron Spray Ionization.

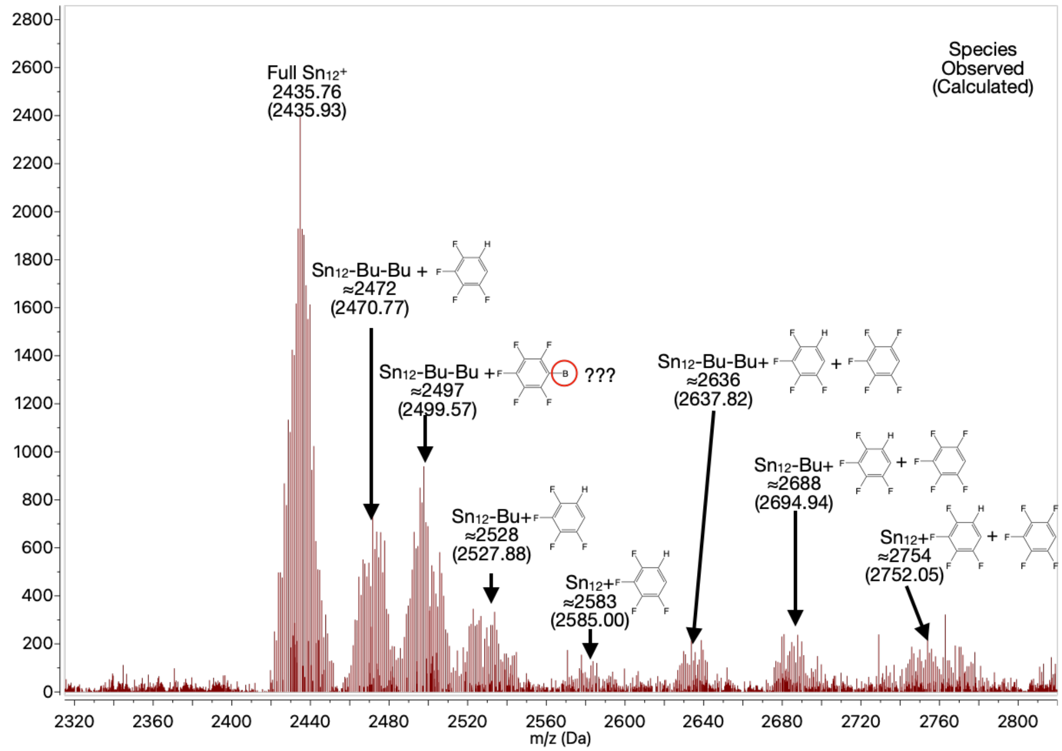

Figure S9: Selected part of mass spectra of positive ions of TinPFPB **1** film exposed to 20 mJ.cm^{-2} of EUV and developed with ethylbenzene obtained via Electron Spray Ionization.

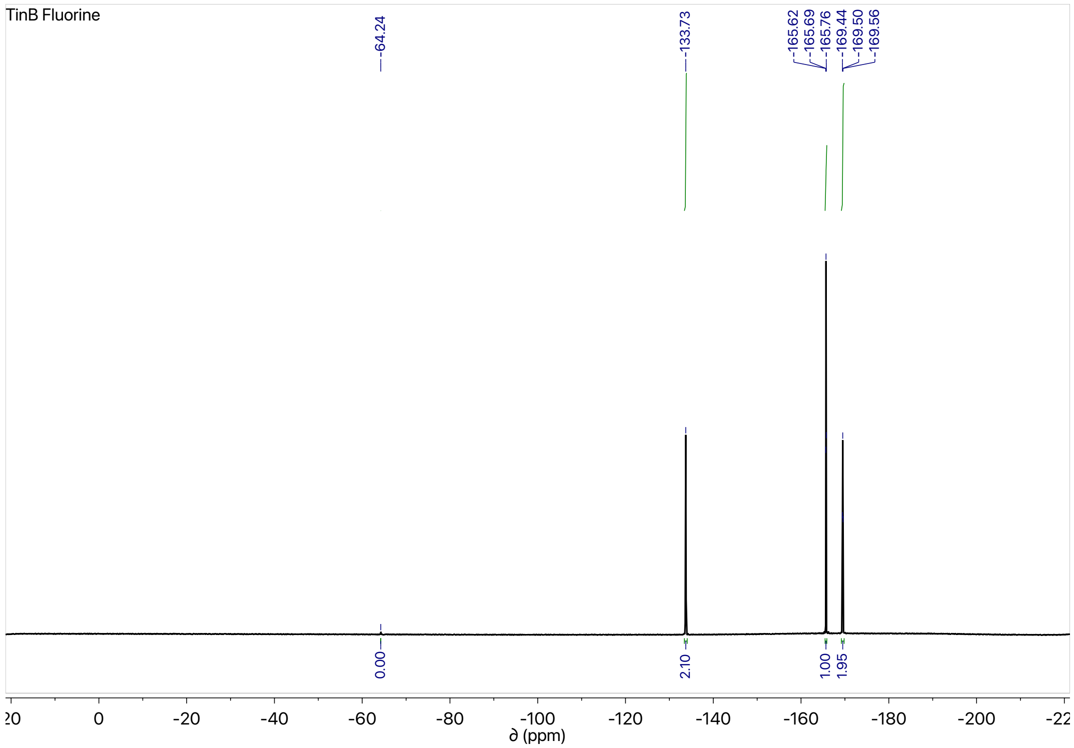

Figure S10: ^{19}F (282.35 MHz, 300 K) NMR spectra of bulk TinPFPB **1** in MeOD.

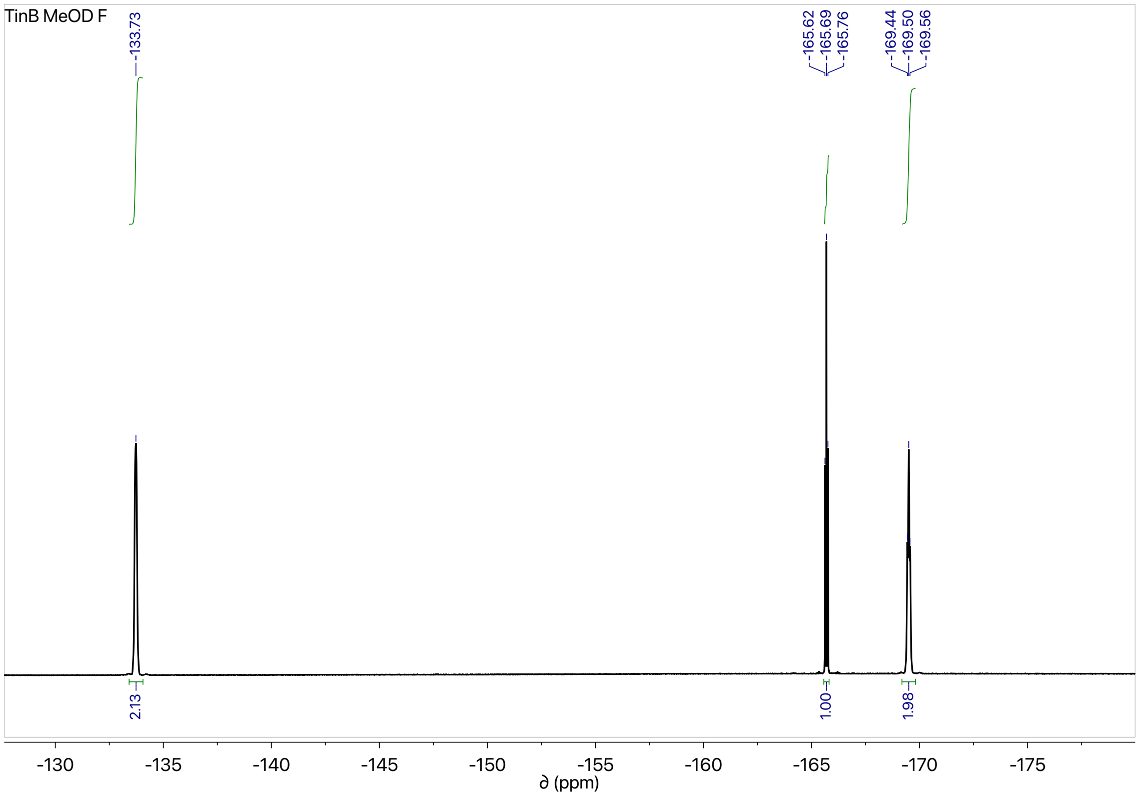

Figure S11: Selected area of ^{19}F (282.35 MHz, 300 K) NMR spectra of bulk TinPFPB **1** in MeOD.

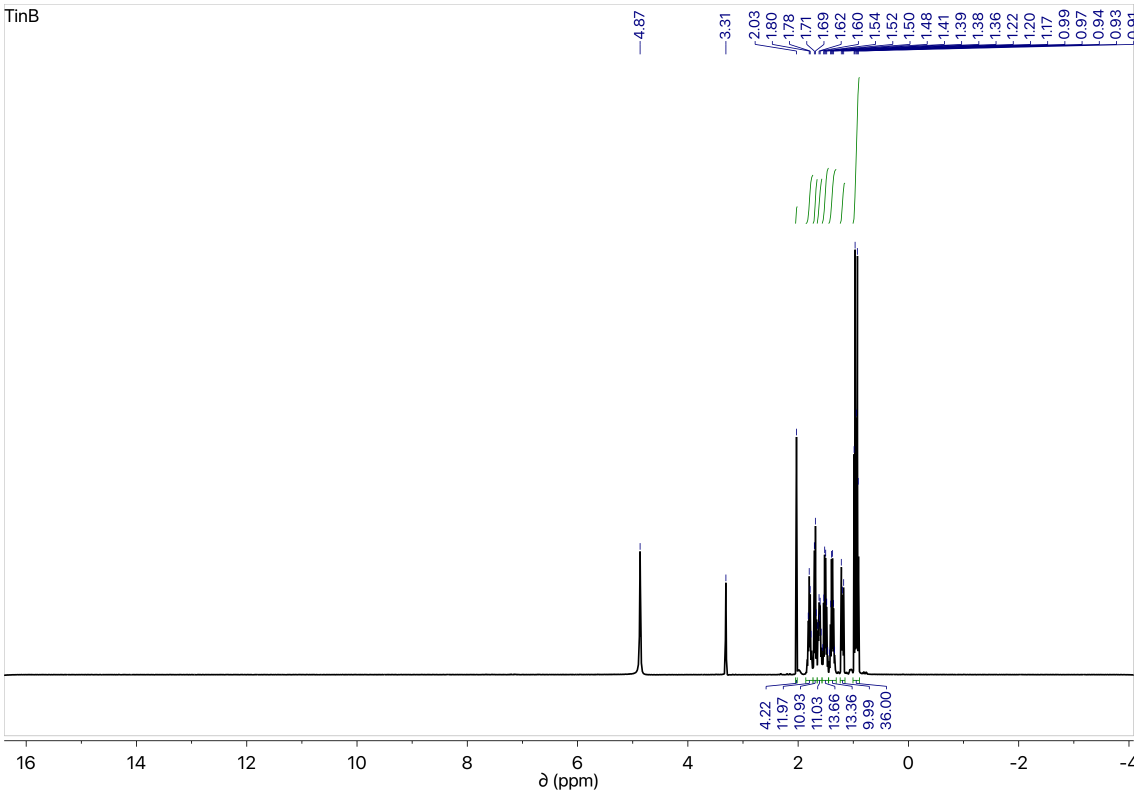

Figure S12: ^{1}H (300 MHz, 300 K) NMR spectra of bulk TinPFPB **1** in MeOD.

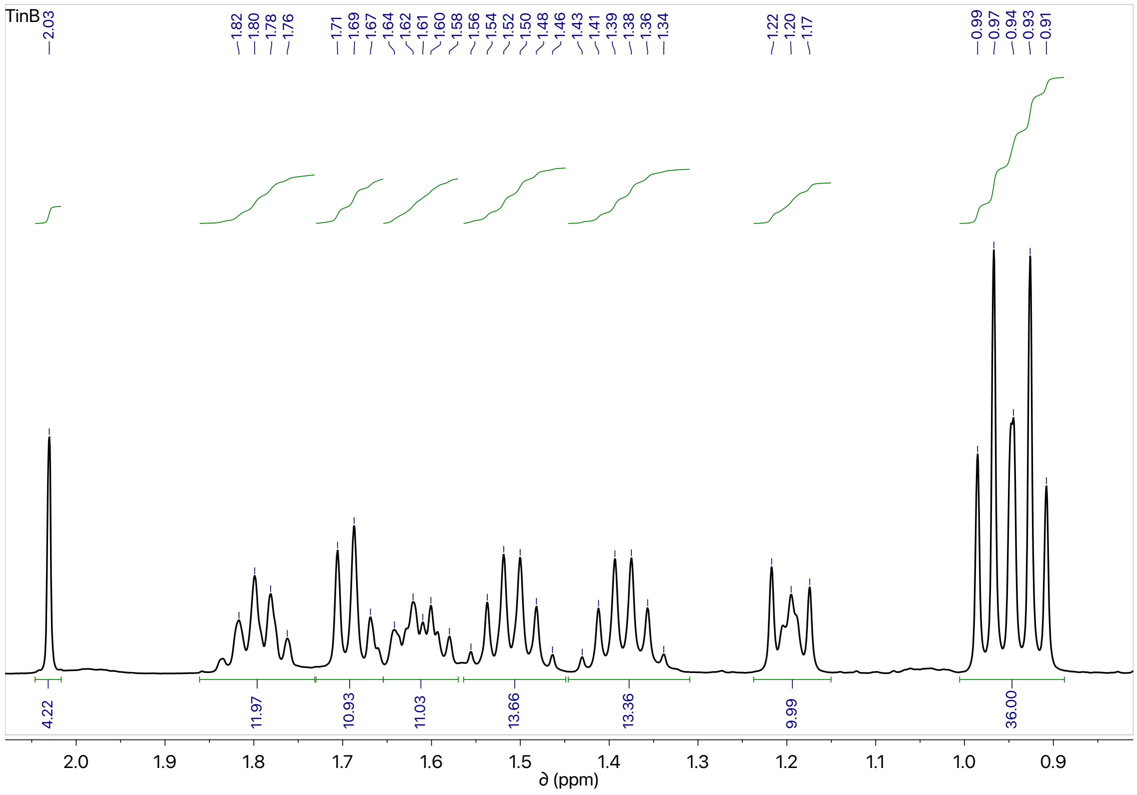

Figure S13: Selected areas of ^{1}H (300 MHz, 300 K) NMR spectra of bulk TinPFPB **1** in MeOD.

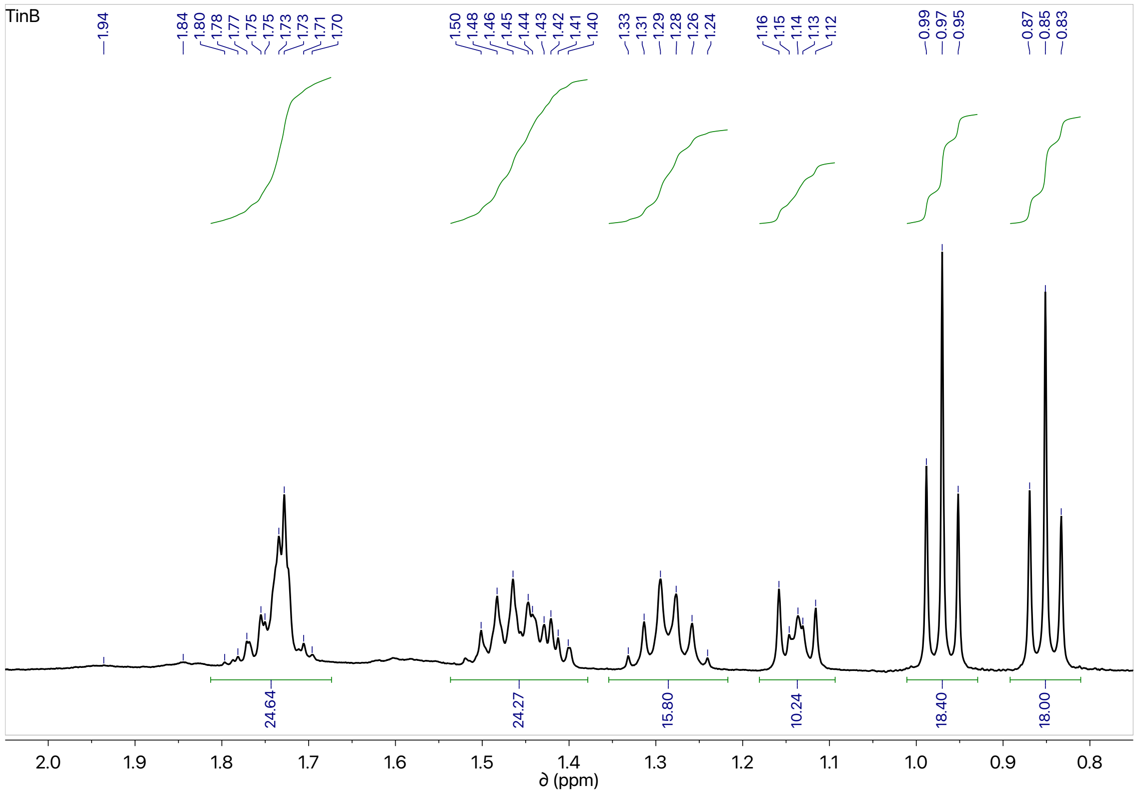

Figure S14: Selected area of ^{1}H (400 MHz, 300 K)NMR spectra of bulk TinPFPB **1** in $CDCl_3$.

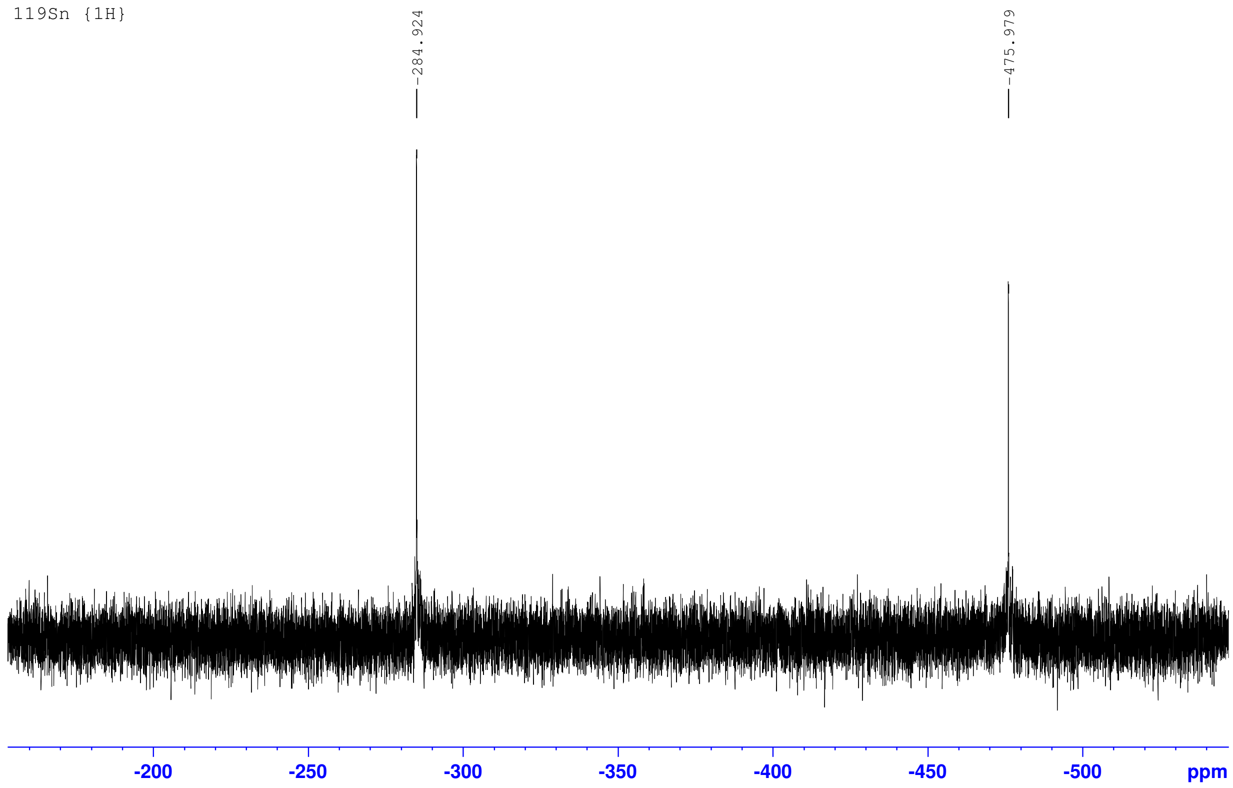

Figure S15: $^{119}Sn\ \{^1H\}$ NMR (149.13 MHz, 300 K) spectra of bulk TinPFPB **1** in MeOD.

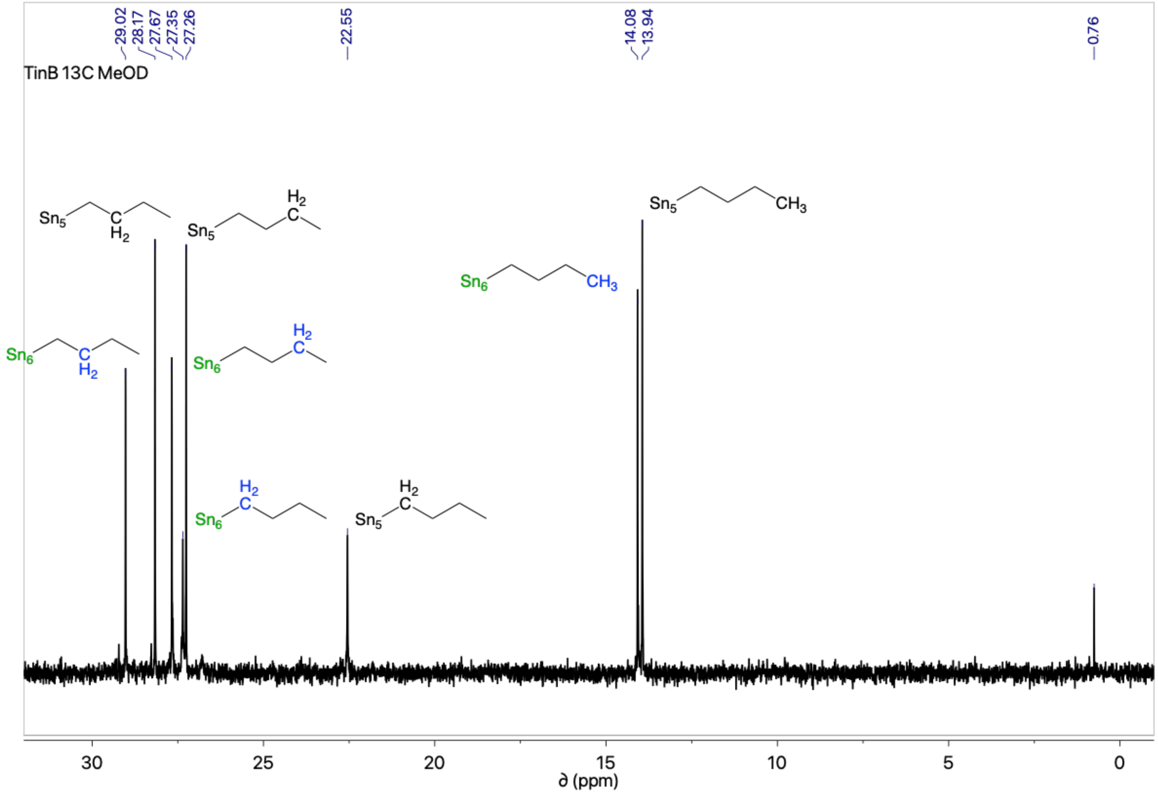

Figure S16: ^{13}C NMR (100.62 MHz, 300 K) spectra of bulk TinPFPB **1** in MeOD.

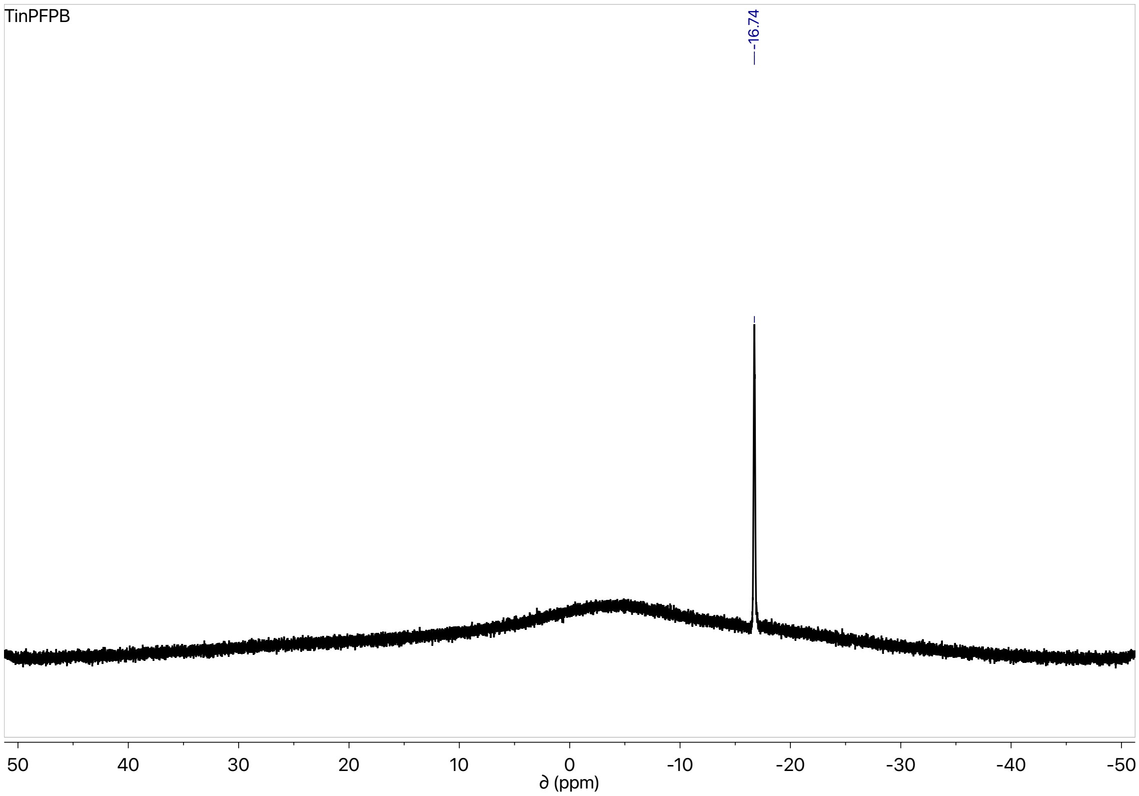

Figure S17: ^{11}B NMR (128.37 MHz, 300K) spectra of bulk TinPFPB **1** in MeOD.

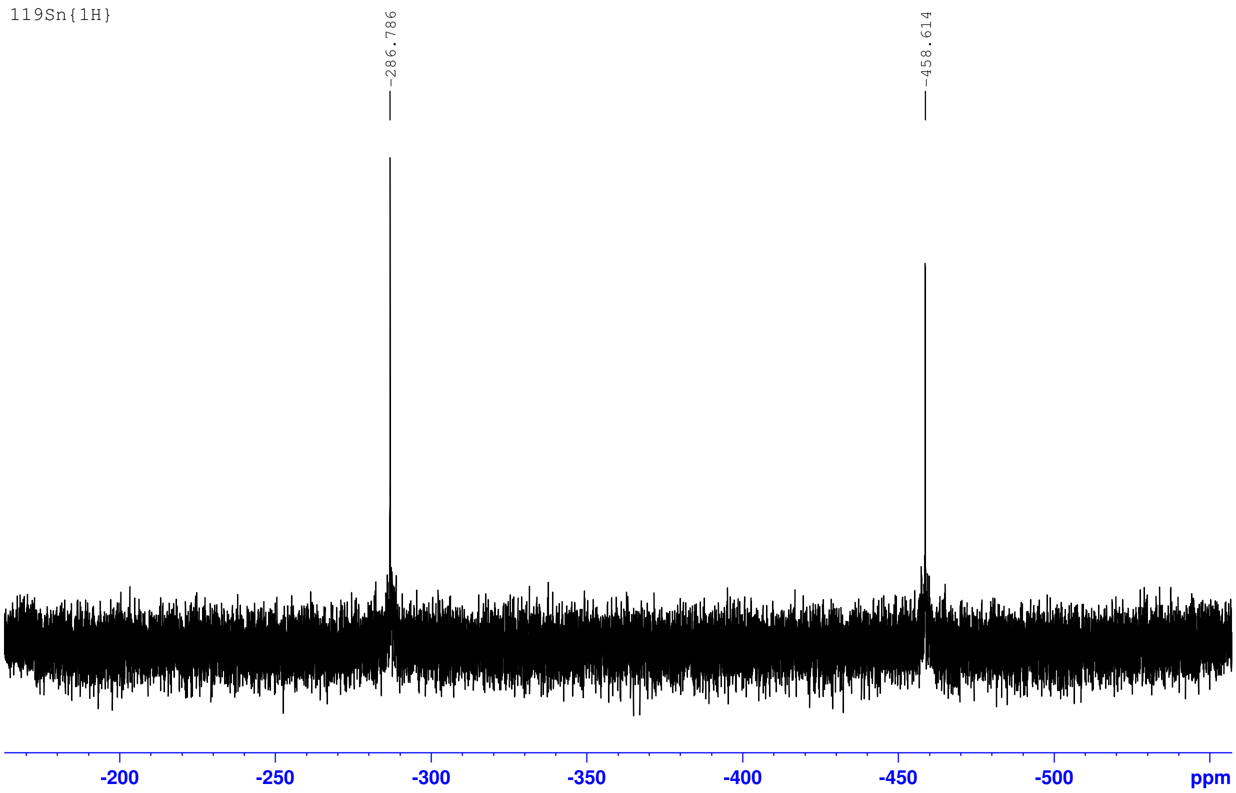

Figure S18: ^{119}Sn {^{1}H} NMR (149.13 MHz, 300 K) spectra of bulk TinTB **2** in MeOD.

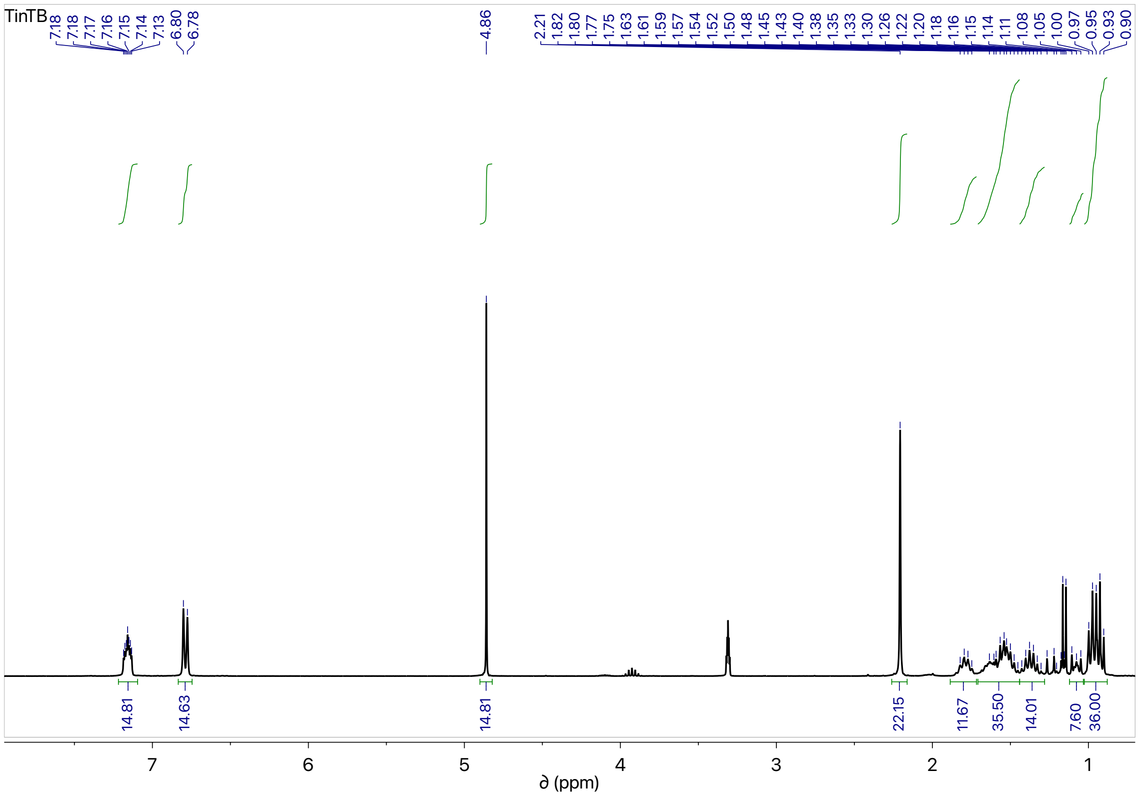

Figure S19: ^{1}H NMR (300 MHz, 300 K) spectra of bulk TinTB **2** in MeOD.

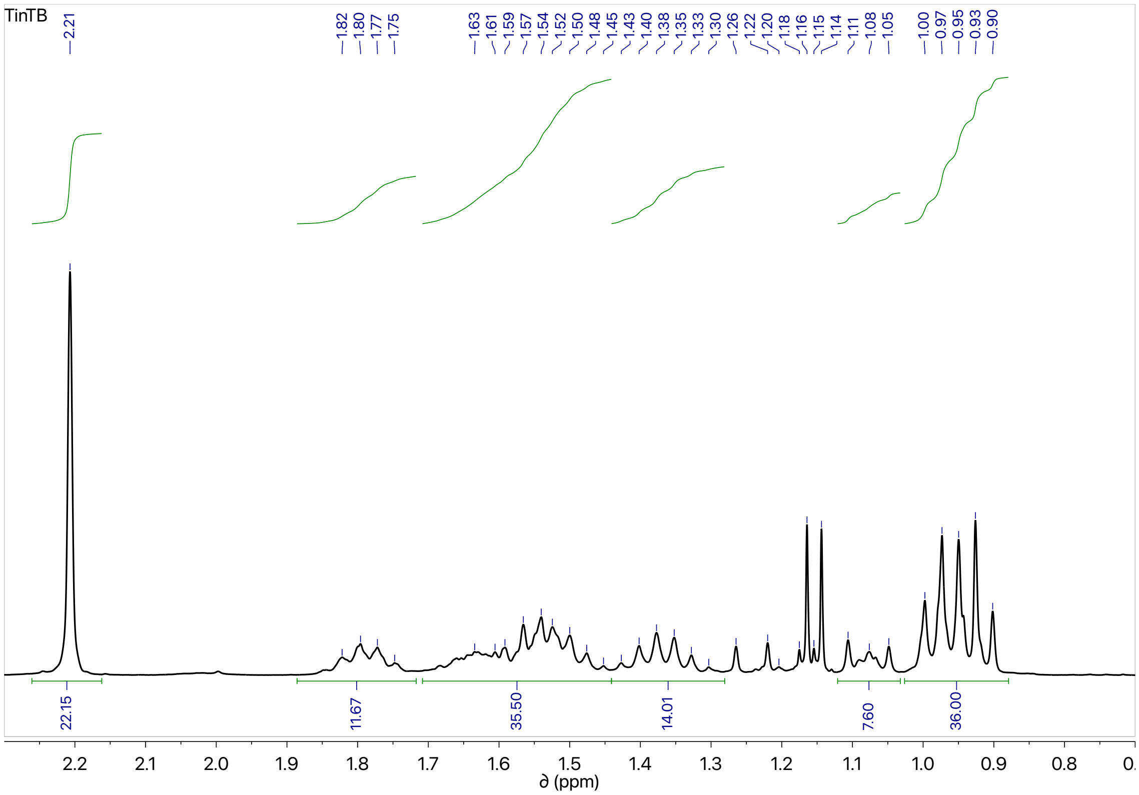

Figure S20: Selected areas of ^{1}H NMR (300 MHz, 300 K) spectra of bulk TinTB **2** in MeOD.

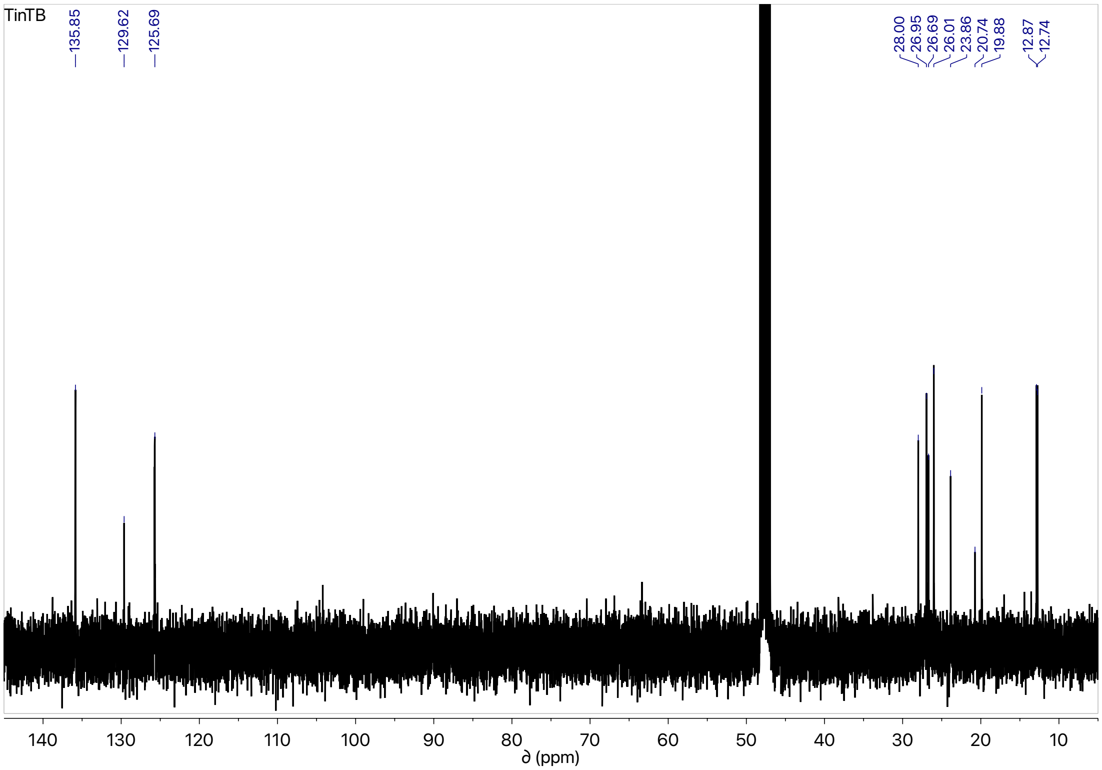

Figure S21: ^{13}C NMR (100.62 MHz, 300 K) spectra of bulk TinTB **2** in MeOD.

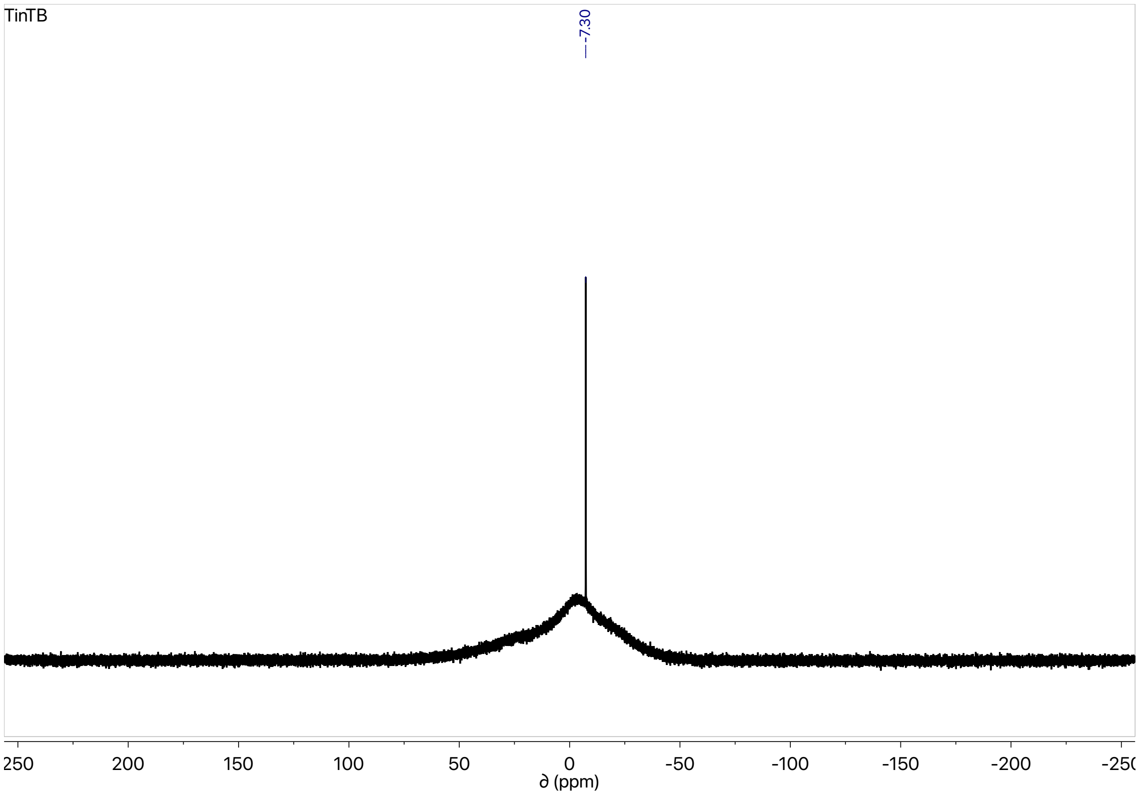

Figure S22: ^{11}B NMR (128.37 MHz, 300K) spectra of bulk TinTB **2** in MeOD.

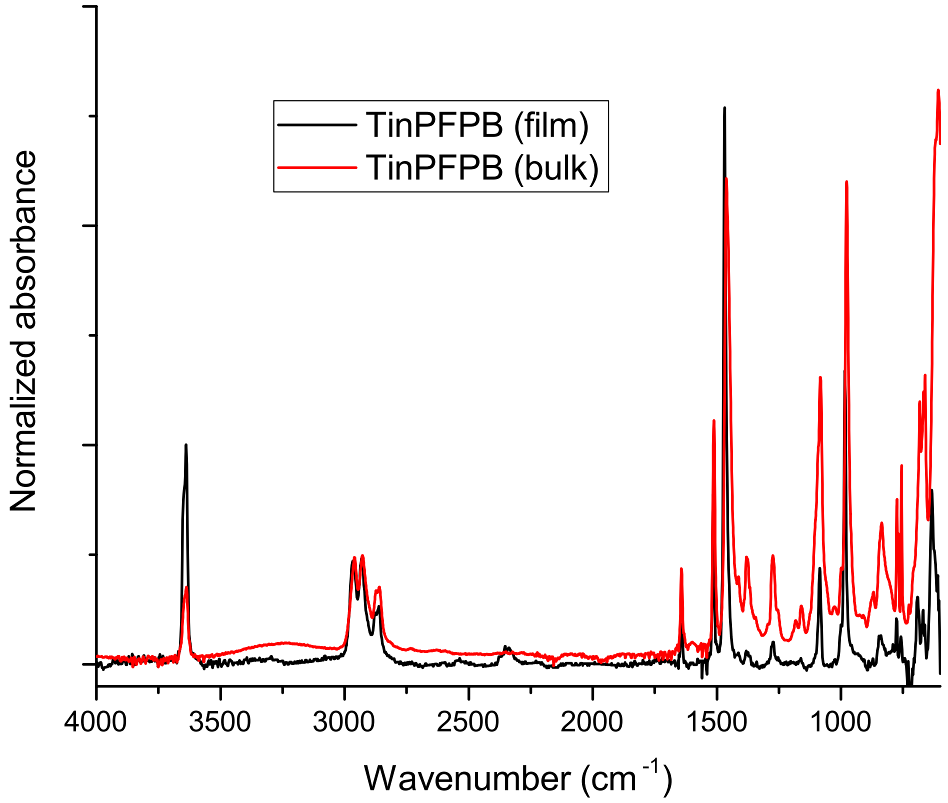

Figure S23: IR spectra of bulk (ATR) and thin film (reflectance) of TinPFPB **1**.

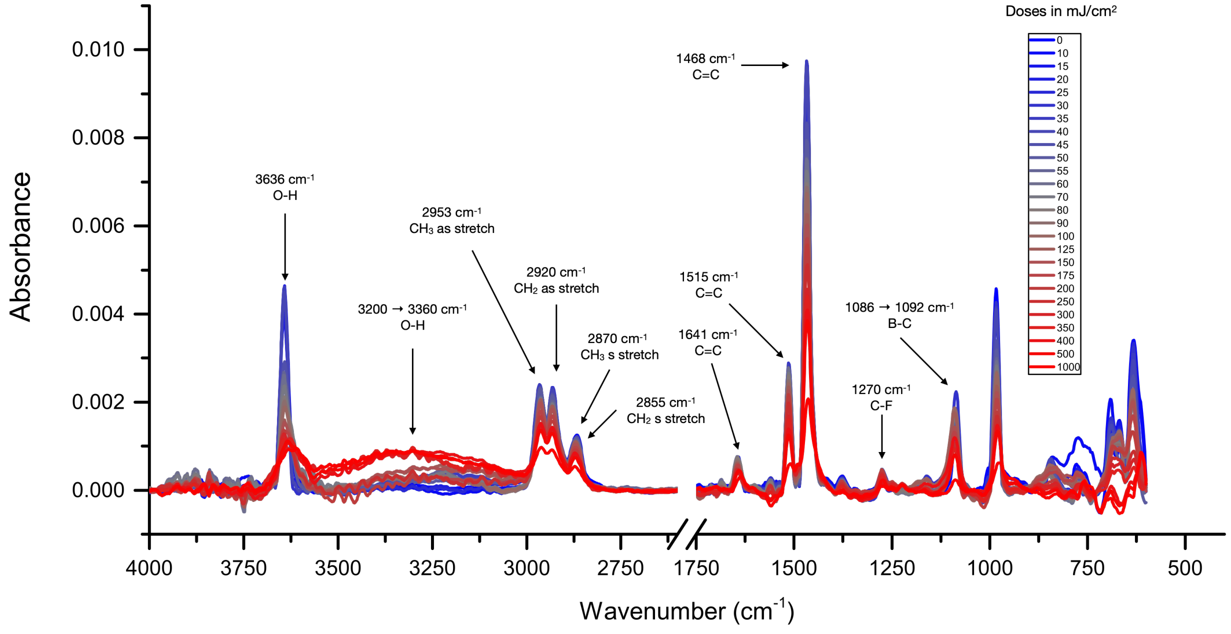

Figure S24: IR spectra of film of TinPFPB **1** exposed to EUV doses ranging from 0 to 1000 mJ.cm^{-2}

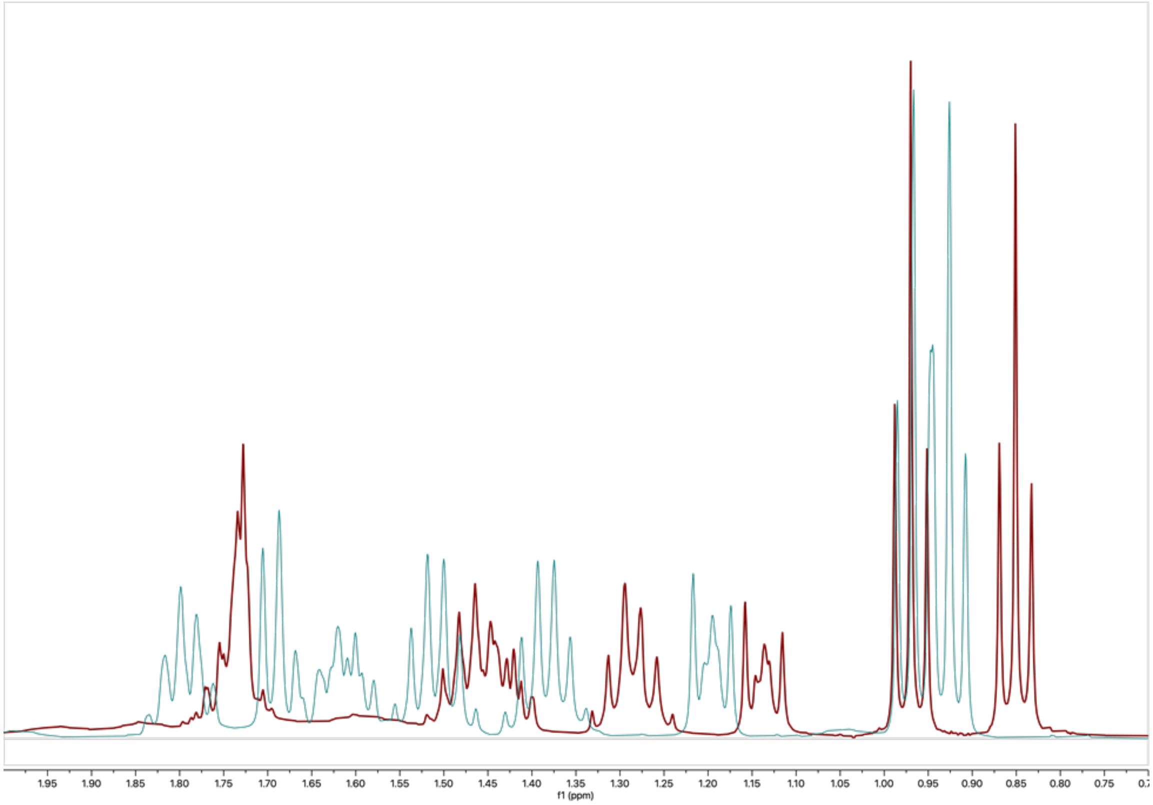

Figure S25: ^{1}H NMR (300 MHz, 300 K) of **1** in MeOD (blue/green) and $CDCl_3$ (brown)

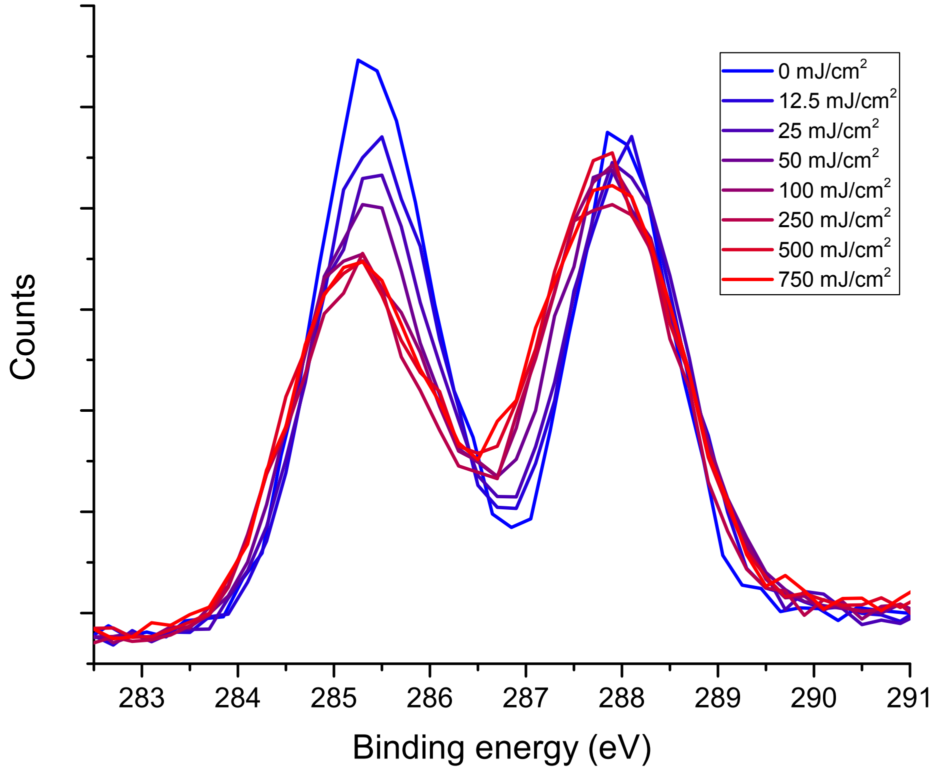

Figure S26: X-ray photoelectron spectra of C1s edge of TinPFPB **1** after in situ 92 eV exposure.

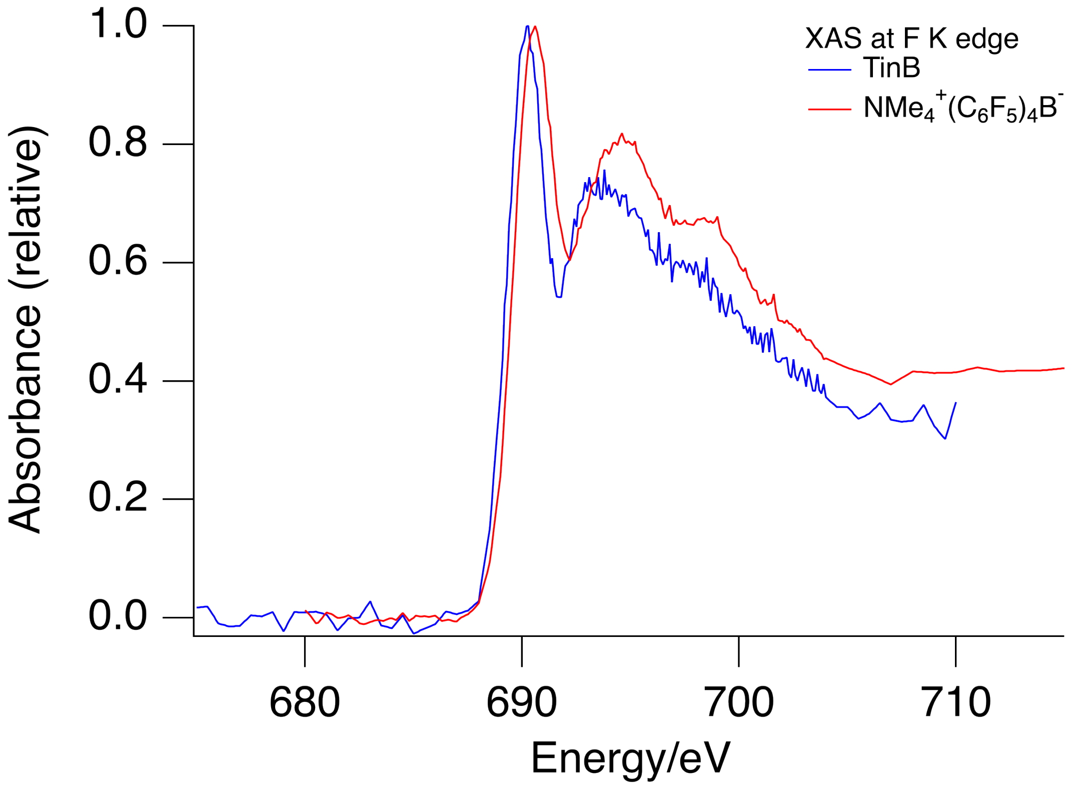

Figure S27: XAS at the F K-edge of **1** (blue) and **4** (red).

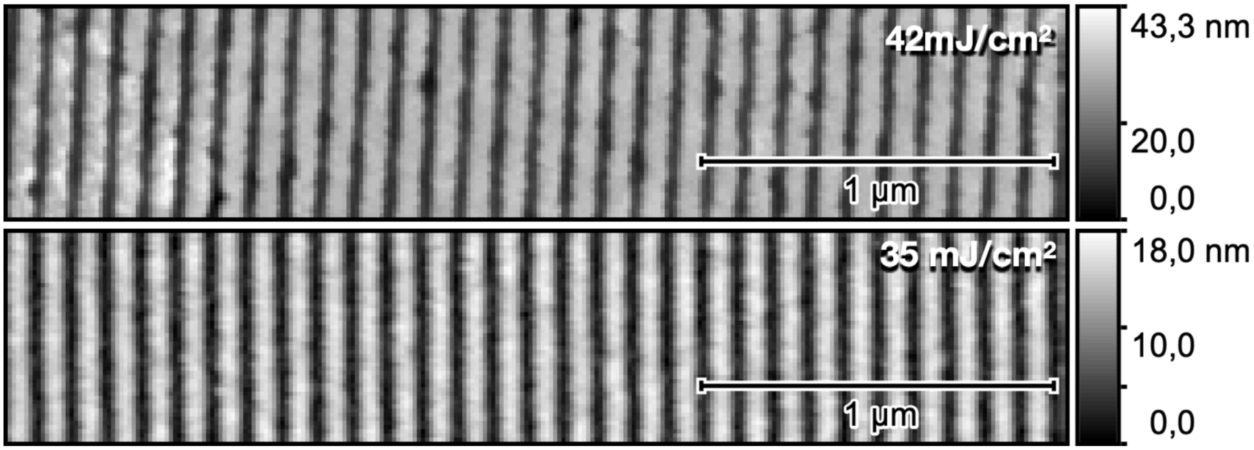

Figure S28: Tapping mode AFM image of TinPFPB (top) and TinTB (bottom) obtained after exposure to 50 nm HP EUV interference lithography and unoptimized development.

11. References

(1) Eychenne-Baron, C.; Ribot, F.; Sanchez, C. New Synthesis of the Nanobuilding Block $\{(BuSn)_{12}O_{14}(OH)_6\}^{2+}$ and Exchange Properties of $\{(BuSn)_{12}O_{14}(OH)_6\}(O_3SC_6H_4CH_3)_2$. *J. Organomet. Chem.* **1998**, *567(1-2)*, 137-142. DOI: 10.1016/s0022-328x(98)00676-7

(2) Bruker, SAINT V8.40B, Bruker AXS Inc., Madison, Wisconsin, USA, 2001.

(3) Krause, L.; Herbst-Irmer, R.; Sheldrick, G. M.; Stalke, D. Comparison of silver and molybdenum microfocus X-ray sources for single-crystal structure determination. *J. Appl. Crystallogr.* **2015**, *48(Pt 1)*, 3-10. DOI: 10.1107/S1600576714022985

(4) Sheldrick, G. M. SHELXT - integrated space-group and crystal-structure determination. *Acta Crystallogr.* **2015**, *71(Pt 1)*, 3-8. DOI: 10.1107/S2053273314026370

(5) Sheldrick, G. M. Crystal structure refinement with SHELXL. *Acta. Crystallogr. C Struct. Chem.* **2015**, *71(Pt 1)*, 3-8. DOI: 10.1107/S2053229614024218

(6) Nannarone, S.; Borgatti, F.; DeLuisa, A.; Doyle, B. P.; Gazzadi, G. C.; Giglia, A.; Finetti, P.; Mahne, N.; Pasquli, L.; Pedio, M.; Selvaggi, G.; Naletto, G.; Pelizzo, M. G.; Tondello, G. The BEAR Beamline at Elettra. *AIP Conference Proceedings* **2004**, *705*, 450-453. DOI: 10.1063/1.1757831

(7) Zhang, Y.; Haitjema, J.; Baljozovic, M.; Vockenhuber, M.; Kazazis, D.; Jung, T. A.; Ekinci, Y.; Brouwer, A. M. Dual-tone Application of a Tin-Oxo Cage Photoresist Under E-beam and EUV Exposure. *J. Photopolym. Sci. Technol.* **2018**, *31(2)*, 249-255. DOI: 10.2494/photopolymer.31.249

(8) Mojarad, N.; Gobrecht, J.; Ekinci, Y. Interference lithography at EUV and soft X-ray wavelengths: Principles, methods, and applications. *Microelectron. Eng.* **2015**, *143*, 55-63. DOI: 10.1016/j.mee.2015.03.047

(9) Raabe, J.; Tzvetkov, G.; Flechsig, U.; Böge, M.; Jaggi, A.; Sarafimov, B.; Vernooij, M. G.; Huthwelker, T.; Ade, H.; Kilcoyne, D.; Tyliszczak, T.; Fink, R. H.; Quitmann, C. PolLux: a new facility for soft x-ray spectromicroscopy at the Swiss Light Source. *Rev. Sci. Instrum.* **2008**, *79(11)*, 113704. DOI: 10.1063/1.3021472

(10) Haitjema, J.; Castellanos, S.; Lugier, O.; Bespalov, I.; Lindblad, R.; Timm, M.; Bülow, C.; Zamudio-Bayer, V.; Lau, J. T.; von Issendorff, B.; Hoekstra, R.; Witte, K.; Watts, B.; Schlathölter, T.; Brouwer, A. M. Soft X-ray Absorption and Fragmentation of Tin-Oxo Cage Photoresists. *Phys. Chem. Chem. Phys.* **2024**, *26(7)*, 5986-5998. DOI: 10.1039/d3cp05428d

(11) Thakur, N.; Vockenhuber, M.; Ekinci, Y.; Watts, B.; Giglia, A.; Mahne, N.; Nannarone, S.; Castellanos, S.; Brouwer, A. M. Fluorine-Rich Zinc Oxoclusters as Extreme Ultraviolet Photoresists: Chemical Reactions and Lithography Performance. *ACS Mater. Au.* **2022**, *2(3)*, 343-355. DOI: 10.1021/acsmaterialsau.1c00059

(12) Yeh, J. J.; Lindau, I. Atomic subshell photoionization cross sections and asymmetry parameters: $1 \leqslant Z \leqslant 103$. *At. Data Nucl. Data Tables.* **1985**, *32(1)*, 1-155. DOI: 10.1016/0092-640x(85)90016-6

(13) Frisch, M. J. T., G. W.; Schlegel, H. B.; Scuseria, G. E.; Robb, M. A.; Cheeseman, J. R.; Scalmani, G.; Barone, V.; Petersson, G. A.; Nakatsuji, H. et al. *Gaussian 16 Revision C.02*; Gaussian, Inc:Wallingford CT: 2016.

(14) te Velde, G.; Bickelhaupt, F. M.; Baerends, E. J.; Fonseca Guerra, C.; van Gisbergen, S. J. A.; Snijders, J. G.; Ziegler, T. Chemistry with ADF. *J. Comput. Chem.* **2001**, *22(9)*, 931-967. DOI: 10.1002/jcc.1056
